# In Vitro Sperm–Epididymosomes Interaction Immediately Before Fertilization Changes Sperm Fertility Potential

**DOI:** 10.1111/andr.70267

**Published:** 2026-05-29

**Authors:** Maíra Bianchi Rodrigues Alves, Maria Alice de Almeida, Ana Beatriz Bossois Moura, Laura Gabrielli Haupenthal, Raissa Braido Rangel, Flávio Vieira Meirelles, Juliano Coelho da Silveira, Felipe Perecin

**Affiliations:** ^1^ Department of Pathology Theriogenology and One Healthy School of Agricultural and Veterinary Sciences São Paulo State University Jaboticabal São Paulo Brazil; ^2^ Department of Veterinary Medicine School of Animal Science and Food Engineering University of São Paulo Pirassununga São Paulo Brazil

**Keywords:** embryo, epididymis, exosomes, IVF, miRNAs, paternal contribution

## Abstract

**Background:**

Sperm acquire fertility ability during epididymal maturation mainly in the epididymal caput and corpus, and once matured, are stored in the epididymal cauda. During storage, interactions with cauda epididymosomes (epEVs) may influence sperm fertility potential; however, the role of such interactions on sperm fertility remains elusive.

**Objectives:**

To investigate the effect of epEVs on sperm fertility potential using the bovine model.

**Materials and Methods:**

A pool of epEVs from cauda epididymal fluid of five bovine males was characterized regarding size, concentration, morphology, and specific markers. To determine the sperm–epEVs interaction protocol, green‐labeled epEVs (PKH67) were incubated with post‐thawed cauda epididymal sperm from three bovine males at ratios of 500, 1000, or 2000 epEVs/sperm, and for incubation periods of 1.5, 3, or 6 h. After that, Hoechst‐stained sperm were analyzed by flow cytometer. Green fluorescence percentage and intensity of 5000 positive Hoechst events were considered as sperm–epEVs interacting. Controls were performed by incubating sperm with PKH67 in PBS. A total of 49 microRNAs (out of 380 investigated) were found detected in the epEVs and the top five were investigated in sperm by quantitative polymerase chain reaction (qPCR) following sperm–epEVs interaction. Subsequently, sperm that interacted with epEVs were used to produce embryos by in vitro fertilization.

**Results:**

epEVs displayed 114.20 ± 3.60 nm, 3.48 × 10^9^ ± 1.84 × 10^8^ particles/mL, a cup‐shaped morphology, and positivity for ALIX, CD‐81, and CD‐63 markers. Following determination of the protocol (1000 epEVs/sperm for 3 h), two microRNAs (miR‐935 and ‐421) previously detected in epEVs, were found in sperm. Finally, sperm incubated with epEVs resulted in higher (p = 0.04) blastocyst rates (epEVs: 38.9% ± 7.3%; 58/149; control: 26.6% ± 5.6%; 40/145).

**Discussion and Conclusion:**

Incubation of sperm with 1000 epEVs/sperm for 3 h promoted sperm–epEVs in vitro crosstalk that enhanced blastocyst rates. These findings suggest that epEVs modulate paternal contribution to development and provide valuable insights to promote a fast and dynamic control of male fertility.

## 1 Introduction

Male infertility accounts for 20%–50% of infertility issues. Out of male infertility causes, 30% remain undiagnosed (reviewed by Boeri et al.) [1] even after investigating hormonal levels, clinical features, and sperm quality. Since male fertility relies on the production of sperm with high fertility potential (i.e., healthy sperm), a proper sperm formation during spermatogenesis is certainly required. However, a healthy sperm is characterized by a sperm with the ability to reach the fertilization site (i.e., ampulla of oviduct), that is, mainly involved with sperm motility; ability to fertilize the oocyte, mainly involved with sperm plasma and acrosome membranes integrity; and ability to contribute to embryo development, such as the presence of sperm‐specific molecules (reviewed by Alves et al.) [2]. Thus, in addition to appropriate formation during spermatogenesis, the acquisition of sperm motility and changes in the sperm molecular profile during epididymal maturation are essential for the fulfillment of male fertility potential.

Throughout maturation in the epididymis, sperm undergo key changes, including the translocation of the protoplasmic droplet from the proximal to the distal position, acquisition of motility, and chromatin stabilization. In parallel, sperm acquire fertilizing capability through dynamic molecular modifications [3, 4, 5, 6, 7, 8]. The epididymis is divided into three segments: caput, corpus, and cauda. In rodents and humans, an initial segment is also included as a fourth segment [5]. Across all segments, the epididymis is a highly coiled duct composed of a pseudostratified epithelium lining a lumen filled with sperm and epididymal fluid [5]. During the 3–10 days of sperm transit through the epididymis in man, mouse, and bovine, sperm undergo deep molecular changes during the passage through the segments of caput and corpus, which last around 0.5–3 days and 0.5–1 day, respectively [5]. Once reaching the cauda segment, sperm display motility and ability to fertilize the oocyte and are stored in this segment until ejaculation. Even though sperm are not predicted to undergo significant molecular changes during storage in cauda, they remain for a longer time in this segment: ∼1.5 in man and ∼6 days in mouse and bovine [5], which corresponds to around 50%–70% of the total period that sperm pass through the epididymis. Since transcriptionally and translationally inert, sperm change their molecular cargo by interacting with the epididymal epithelial cells along the epididymal transit. Even in a minor proportion, it is expected that sperm change their molecular cargo and fertility potential also during storage in epididymal cauda.

Depending on the epididymal segment, the proportion of the types of epithelial cells varies. In epididymal cauda, principal cells are predominant, similar to the other segments; however, there is a higher proportion of clear cells in comparison with the other segments [5]. Both types of cells interact with sperm in different forms. In that regard, principal cells interact directly with sperm by stereocilia [9], characterizing a physical interaction. Recently, nanotubes of clear cells [10] were also described as a possible mechanism of direct interaction. However, the indirect interactions characterized mainly by sperm interaction with luminal extracellular vesicles (EVs) [11] are the major mechanism of sperm interaction with epididymal epithelial cells. Out of EVs nanoparticle types (i.e., exosomes, microvesicles, and apoptotic bodies), exosomes are small EVs of around 30–150 nm of diameter composed of a lipid layer originated from a multivesicular endosome of a somatic cell that transport membrane and cytosolic lipids, proteins, and RNAs [12]. Particularly on the epididymis, the small EVs display a diameter lower than 200 nm and are referred to as epididymosomes (epEVs) being released mainly by principal cells to the epididymal lumen [13]. Recently, epEVs were also described as probably released by clear cells [10]. Although there are different types of epEVs, evidence clearly indicates that epEVs interact with sperm, deliver key molecules as RNAs and proteins to sperm, and participate in the sperm maturation process [14].

epEVs are released in the luminal fluid of the epididymis and interact with sperm, modulating the molecular cargo of sperm cells [14]. The molecular content of epEVs varies according to the epididymal segments, thus, epEVs isolated from epididymal caput display a different molecular content compared to epEVs isolated from epididymal cauda [11, 15]. In that regard, miR‐395, ‐654, and ‐1224 were found more abundant in epEVs from bovine epididymal cauda [11, 15]. On the other hand, bovine epEVs isolated from epididymal caput and cauda showed similar cargo [15] of microRNAs (miRNAs) involved in sperm contribution to embryo development, such as miR‐449 in bovine [16], miR‐34c in mouse [17], and miR‐191 in human [18]. The consistent presence along epididymis might be required to guarantee that these key miRNAs are certainly acquired by sperm. In parallel, mouse sperm isolated from epididymal caput and cauda display different miRNAs cargo [4] indicating a modulation of sperm content during epididymal transit. In addition, other molecules associated with epEVs, such as proteins and circRNAs, are also involved in sperm maturation [19]. Also, experimental sets in mice indicated that embryos produced with sperm from caput supplemented with RNAs isolated from cauda displayed higher preimplantation and postimplantation rates compared to embryos produced with only sperm from caput [20]. This study clearly demonstrated the importance of molecules contained in the epididymis cauda for determining sperm fertility potential.

Although most changes in sperm molecular cargo occurs in the caput [4], sperm remain stored for a long period in the epididymal cauda. Thus, the interaction between cauda epEVs and sperm might lead to changes in sperm molecular cargo. In addition, sperm‐borne molecules critical to embryo development are present in epEVs from cauda (called here as epEVs), such as the miR‐34c [15]. Hence, sperm–epEVs interaction in the context of epididymal cauda potentially modulates sperm fertility potential. Therefore, the aim of this study is to investigate in vitro the effects of incubation of cauda epididymal sperm with cauda‐derived epEVs on sperm fertility potential. To this end, a model of fast and dynamic interaction was validated to determine the ratio of epEVs per sperm and the period of coincubation to obtain significant interaction between sperm and epEVs using the bovine as a model. Then, after sperm–epEVs interaction, sperm cells were assessed for the presence of epEVs’ miRNAs. Finally, the fertility potential of epEV‐interacted sperm was determined by embryo production via in vitro fertilization (IVF). In summary, we demonstrated that sperm interact with epEVs in an in vitro model based on cauda microenvironment, and that this interaction modulates sperm fertility potential by changing the blastocyst rates. The data presented here provide valuable insights regarding the mechanisms involved in the modulation of sperm fertility potential immediately before fertilization.

## 2 Materials and Methods

### 2.1 Reagents and Ethics

All the materials were purchased from Sigma–Aldrich unless otherwise stated. The Ethics Committee on Animal Use of the School of Animal Science and Food Engineering of the University of São Paulo (CEUA/FZEA) approved the present experiment by protocol number 8312020920.

### 2.2 Experimental Design

Epididymal fluids isolated from the cauda of five bulls were collected to form a pool of epEVs. After isolation, epEVs were assessed regarding size, concentration, morphology, and specific markers. Once characterized, epEVs were labeled with a green marker (PKH67) and incubated with post‐thawed epididymal sperm to verify the interaction between sperm and epEVs. For the incubation of sperm and epEVs, motile sperm were selected by a Percoll gradient and 1 × 10^6^ sperm/mL were incubated with epEVs as follows: 500 (0.5×), 1000 (1×), or 2000 (2×) epEVs/sperm. All treatments were tested in three incubation times: 1.5, 3, or 6 h. At the end of each period, the solution was centrifuged (600 *g*/5 min), and the sperm pellet was resuspended in TALP‐sperm and analyzed on flow cytometer system and confocal to determine the occurrence of sperm and green epEVs interaction. Control was performed with sperm incubated with PBS added with the green marker and with no epEVs for all treatments. Once the concentration of epEVs/sperm and period of interaction was determined, the protocol was used to verify the presence in sperm of epEVs miRNA molecules and sperm fertility potential in which two groups were tested: (1) control—sperm not incubated with epEVs, and (2) epEVs—sperm incubated with EVs. For that, the miRNA profile of 380 miRNAs of the epEVs was determined. Following that, the top five abundant miRNAs found in epEVs were investigated in sperm after interaction with epEVs to verify changes in sperm molecular profile. The miRNA investigation was performed by quantitative polymerase chain reaction (qPCR). For sperm fertility potential, after incubating sperm with epEVs, sperm were used to produce embryos by IVF. Rates of first cleavage, cleavage, and blastocyst were evaluated. For all experiments, cryopreserved epididymal sperm were obtained from the epididymal cauda of three bulls, forming three epididymal cauda sperm batches. Sperm batches were cryopreserved according to the protocol described in De Almeida et al. [21] using BoviFree extender (Minitube, Germany) and 2.5 h of equilibrium time.

### 2.3 Obtention of Epididymal Fluid to Isolation of epEVs and Sperm

For the collection of epididymal fluid, male reproductive tracts were collected from a slaughterhouse at 4°C. At the laboratory, epididymis was separated from testis and washed with saline solution (NaCl 0.9%), added with 500 IU/mL of penicillin and 500µL/mL of streptomycin. The epididymal connective tissue was carefully removed, and epididymal cauda was isolated from epididymal corpus, preserving the deferent duct (Figure S1). All the procedure was performed at 4°C. After that, the epididymal fluid was collected by washing the cauda lumen with 10 mL of PBS (phosphate buffered saline) (8 mg/mL NaCl, 0.2 mg/mL KCl, 1.44 mg/mL Na_2_HPO_4_, and 0.24 mg/mL KH_2_PO_4_) using the retrograde flow technique based on Belleannée et al. [15]. For that, a catheter of 22 G coupled to a 10 mL syringe was introduced via deferent duct as shown in Figure S2 and Video S1. At the end of the process, the epididymal fluid from both cauda epididymides of each bull was collected into a single 50‐mL tube. The epididymal fluid was kept at 4°C until epEVs isolation or sperm cryopreservation.

### 2.4 Isolation of epEVs From Epididymal Fluid

The isolation of epEVs was performed from the epididymal fluid by ultracentrifugation according to Alves et al. [22]. For this, the epididymal fluid was first centrifuged at 600 *g* for 10 min to remove the cells. After that, the supernatant was centrifuged at 4000 *g* for 20 min twice. Finally, centrifugation was performed at 16,500 *g* for 30 min and the supernatant was kept at −80°C. Then, the supernatant was ultracentrifuged at 120,000 *g* for 70 min each. All centrifugations were performed at 4°C. In the end, the epEV pellets were diluted in PBS and analyzed for the characteristics of the diameter and the concentration, morphology, and specific markers. After characterization, epEVs were isolated for staining and to test the interaction with sperm. Also, for each experimental set, epEVs were isolated to be incubated with sperm before molecular analysis and embryo production. For all experimental sets, epEVs were used fresh, that is, EVs were used immediately after isolation by ultracentrifugation.

### 2.5 Diameter and Concentration of epEVs

For the analysis of the diameter and concentration, the NanoSight NS300 and NanoSight software 3.4.4 (Malvern Panalytical, Malvern, UK) were used. For that, 1 mL of the epididymal fluid was ultracentrifuged to isolate epEVs that were diluted in 95 µL of PBS. Then, an aliquot of 5 µL of the epEVs was diluted in 995 µL of PBS and inserted in a device that was programmed to record the particles in five videos of 30 s. The camera was programmed for Level 14, Gain 2, and the reading took place at a temperature of 38.5°C. As a standard, the device was previously calibrated with beads of known size (50, 100, and 150 nm; Beads, Malvern, UK). Finally, the concentration of particles and the mode of the diameter of the particles were quantified.

### 2.6 Morphology of epEVs

For the evaluation of morphology of epEVs, transmission electron microscopy (TEM) was used. Following the isolation of epEVs, the supernatant was discarded, and the pellet was resuspended in 50 µL of PBS, and 200 µL of fixative solution (0.1 M cacodylate, 2% glutaraldehyde, and 2% paraformaldehyde at pH 7.2–7.4) was added and kept for 2 h at room temperature. Finally, to remove the fixative solution, 2 mL of PBS was added to each sample, and ultracentrifugation was performed using the same parameters mentioned above. Afterward, the supernatant was discarded, and the pellet was resuspended in 50 µL of buffer solution (1% cacodylate). Then, 6 µL of the sample was placed on a copper grid coated with pioloform and kept for approximately 15 min to air dry at room temperature. Finally, 2% uranyl acetate was added to the grid for 3 min, the excess was removed with filter paper, and the samples were analyzed under a transmission electron microscope (FEI Tecnai 20; emission LAB6; 200 kV).

### 2.7 Specific Markers of epEVs

Specific markers of epEVs were evaluated by flow cytometric analysis. For that, epEVs were isolated and labeled with specific antibodies. For this, the antibodies CD81‐PE (Abcam, UK; cat. AB81436), ALIX‐PE (Santa Cruz Biotechnology, USA; cat. sc‐53540), and CD63‐FITC (Abcam, UK; cat. AB18235) were used. After epEVs isolation, 10 µL of PBS was added to 10 µL of the isolated sample, then 1 µL of CD81‐PE antibody was added, and the sample was kept at room temperature for 2 h. For the ALIX‐PE antibody, since it is an intracytoplasmic protein, prior to coincubation, 10 µL of the isolated epEVs were treated with 10 µL of 0.001% Triton X‐100 solution (X100, Sigma–Aldrich) for 15 min at room temperature for permeabilization. Subsequently, 20 µL of PBS was added to the solution to stop the Triton X‐100 reaction. After that, 1 µL of ALIX‐PE was added and coincubated for 1 h at room temperature. For the CD63‐FITC antibody, 10 µL of the isolated epEVs was mixed with 1 µL of the antibody and coincubated at room temperature for 2 h. Finally, the mitochondrial membrane marker TOM20 (Santa Cruz Biotechnology, USA; cat. SC‐17764) was used as a negative marker to demonstrate the absence of contaminants in the isolated epEVs. For this, 50 µL of the isolated epEVs was subjected to the permeabilization protocol with Triton, as previously described. Afterward, the sample was mixed with 1 µL of TOM20 antibody and coincubated at room temperature for 1.5 h. For TOM20, sperm was used as a positive control (Table S1). At the end of the coincubation periods, 200 µL of PBS was added to each sample and analyzed using CytoFLEX (Beckman Coulter, USA) and the CytoExpert software (Beckman Coulter, USA). The population of nanoparticles ranging from 100 to 300 nm in size was considered for analysis. To this end, gain and threshold settings were adjusted according to the manufacturer's recommendations using fluorescent Megamix‐Plus SSC and FSC beads (BioCytex, France), covering sizes from 100 to 900 nm and the fluorescence of interest. Prior to sample acquisition, antibodies in the absence of epEVs were analyzed. For this purpose, PBS containing antibody was used as a negative control (no epEVs). Each antibody was incubated with PBS following the same protocol applied to epEVs. After incubation, the gating was adjusted to ensure that nanoparticles corresponding to free antibodies were excluded from sample analysis. Finally, samples were analyzed once based on the number of events within the defined gating.

### 2.8 Labeling of epEVs

The epEVs were labeled with PKH67 Green Fluorescent Cell Linker to investigate the interaction with sperm. For that, following isolation, epEVs were resuspended in 95 µL of PBS, from which an aliquot of 5 µL diluted in 995 µL of PBS was removed for analysis of concentration and size in NanoSight NS300 and NanoSight software 3.4.4 (Malvern Panalytical, Malvern, UK). The volume of 90 µL containing the necessary quantity of nanoparticles to incubate with sperm was stained with 6 µL of PKH67 solution in 750 µL of Diluent C for 15 min at 38.5°C. At the end of the incubation, the dye was inactivated by the addition of 1% bovine serum albumin (BSA) in PBS for 1 min at room temperature. Afterward, 1 mL of PBS was added, and the labeled epEVs were isolated by ultracentrifugation at 100,000 *g* for 30 min at 4°C. Afterward, the sample of labeled epEVs was resuspended in 90 µL of PBS, and the labeled epEVs were incubated with sperm. The same staining protocol was performed with 90 µL of PBS, which was stained with PKH67 solution, ultracentrifuged, and resuspended in 90 µL of PBS and used as a negative control.

### 2.9 Interaction of epEVs and Sperm

To perform coincubation of epEVs and sperm, after labeling epEVs with PKH67 Green Fluorescent Cell Linker, epEVs were incubated with sperm at different concentrations (500 epEVs/sperm, 1000 epEVs/sperm, or 2000 epEVs/sperm, respectively; 0.5×, 1×, and 2× treatments) during different periods (1.5, 3, and 6 h). For this, cryopreserved straws from epididymal cauda sperm were thawed (37°C/30 s). After that, motile sperm were selected by a Percoll gradient and 1 × 10^6^ sperm/mL were incubated in IVF medium (Tyrode's lactate stock supplemented with 50 µg/mL gentamicin, 0.2 mM sodium pyruvate, 18 mM penicillamine, 10 µM hypotaurine, 1.8 µM epinephrine, 10 µg/mL heparin, and 6 mg/mL BSA) with epEVs to perform 0.5×, 1×, and 2× treatments. The samples were incubated in a four‐well plate with 400 µL of IVF medium in 5% of CO_2_, at 38.5°C in air for 1.5, 3, or 6 h. At the end of the incubation period, the solution was centrifuged (600 *g*/5 min) to isolate the sperm pellet that was resuspended in Talp‐Sperm (4.2 mg/mL NaCl, 1.87 mg/mL KCl, 2.1 mg/mL NaHCO_3_, 0.05 mg/mL NaH_2_PO_4_, 0.145 mg/mL CaCl_2_H_2_O, 0.08 mg/mL MgCl_2_6H_2_O, 6.5 mg/mL HEPES, supplemented with 5 mg/mL glucose, 18.50 mL sodium lactate, 140 mg/mL sodium pyruvate, and 200 mg/mL BSA) to a concentration of 5 × 10^6^ sperm/mL. Sperm were stained with 1 µL of Hoechst 33342 0.05 mg/mL and analyzed on CytoFLEX System B3‐R0‐V3flow cytometer system and CytoExpert software (Beckman Coulter, USA), equipped with lasers emitting at 375, 405, 488, and 638 nm and 15 optical filters for evaluation of sperm that interacted with green epEVs. For that, a total of 5000 positive events for Hoechst were considered as sperm, and those with higher green fluorescence intensity were considered the percentage of sperm that interact with epEVs. The median of the fluorescence intensity per sperm was also used to evaluate the sperm–epEVs interaction. Control was performed with sperm incubated with PKH67 in PBS for all treatments. For visualization of labeled epEVs in sperm, acquisition of images was performed in fluorescence microscopy. In addition, a real‐time imaging using time‐lapse was performed assessing a total of 1000 epEVs/sperm under Thunder Imager 3D Assay (Leica, Germany) fluorescence microscopy with objective of 63×. For this purpose, the Leica LAS X software was used and configured to acquire images at 4‐s intervals, generating consecutive 15‐min videos over a total observation period of 3 h to monitor sperm–epEVs interaction.

### 2.10 Detection of miRNAs in epEVs and Sperm by qPCR

To investigate miRNAs in epEVs and sperm, first, a profile of 380 miRNAs was investigated in epEVs (Tables S2 and S3). For this, RNA was extracted from approximately 7.5 × 10^9^ epEVs using miRNeasy Mini Kit (Qiagen, Germany), according to the manufacturer's recommendation. Total RNA was quantified by NanoDrop 2000 spectrophotometer (Thermo Fisher Scientific, USA). cDNA synthesis and quantitative RT‐PCR were performed as described by Ferst et al. [23]. Briefly, 100 ng of total RNA was used for cDNA synthesis in a total volume of 10 µL. Subsequently, quantitative RT‐PCR was carried out using GoTaq qPCR Master Mix (Promega, USA), combined with 10 µL of cDNA, universal primer (10 µM), and specific forward primer (10 µM). The expression profile was analyzed in 384‐well plates using QuantStudio 6 Flex Real‐Time PCR System (Thermo Fisher Scientific, USA). The PCR cycling conditions were initial incubation of 95°C for 5 min, followed by 45 cycles of 95°C for 10 s, 60°C for 30 s, and 70°C for 30 s. The melting curve (95°C for 15 s, 60°C for 1 min, and 95°C for 15 s) was generated to confirm amplification specificity. Reactions with quantitative cycles (Cq) greater than 37 were excluded according to Bustin et al. [24]. For normalization, Hm/Ms/Rt T1 snRNA was used as the endogenous control for epEVs. Relative transcript levels were calculated using the comparative Cq method and transformed by the 2^−∆Cq^ for graphical representation [25].  After determining the abundance of miRNAs in epEVs, the five most highly expressed miRNAs were further investigated in sperm. For this purpose, sperm from the cauda of the epididymis were coincubated with epEVs for 3 h (1 × 10^6^ sperm/mL). After coincubation with epEVs, sperm were collected and centrifuged at 600 *g*/5 min twice to remove free epEVs. Next, RNA was extracted using RNAzol (R4533, Sigma–Aldrich, USA). Reverse transcription and quantitative polymerase chain reaction (RT‐qPCR) were performed according to the protocol described by Ferst et al. [23]. A total of 300 ng of RNA was used for the cDNA stage. Afterward, 10 µL of cDNA was used to prepare the RT‐qPCR mix, and RT‐qPCR was performed under the same conditions as previously described. Data were normalized using miR‐99b. Finally, the data were transformed using 2^−∆Cq^ [26]. For functional enrichment analysis, the predicted target transcripts of the miRNAs identified in epEVs‐interacted sperm were obtained using the TargetScanHuman platform (https://www.targetscan.org/vert_80/). After that, all transcripts were uploaded to the DAVID Bioinformatics Resources (NIAID/NIH; https://davidbioinformatics.nih.gov) for KEGG pathway analysis. The signaling pathways were ranked according to their associated *p*‐values.

### 2.11 Embryo In Vitro Production

Ovaries were collected from a slaughterhouse, and ovarian follicles (3–6 mm) were aspirated with 18 G needles in the laboratory. The evaluation of cumulus–oocyte complexes (COCs) was performed under a stereomicroscope (SMZ 745T model, Nikon, Tokyo, Japan). Then, COCs were washed in tissue culture medium 199 (TCM 199, Gibco, Thermo Scientific) supplemented with 10% fetal bovine serum (FBS), 22 µg/mL sodium pyruvate, and 50 µg/mL gentamicin. Only Grade I and Grade II COCs were selected and washed in maturation medium (TCM 199 supplemented with 26 mM sodium bicarbonate, 10% FBS, 22 µg/mL sodium pyruvate, 50 µg/mL gentamicin, 0.5 µg/mL follicle‐stimulating hormone [FSH] [FolltropinTM, Bioniche Animal Health, Belleville, Canada], and 5 IU/mL human chorionic gonadotropin [hCG] [VetecorTM, Hertape Calier, London, England]). A total of 20–25 COCs were placed in 90 µL of maturation medium covered with mineral oil for 22–24 h. After the period, oocytes were coincubated for 6 h with epididymal sperm from control or epEVs (previously incubated with epEVs) and incubated in IVF medium composed of Tyrode's lactate stock supplemented with 50 µg/mL gentamicin, 22 µg/mL sodium pyruvate, 40 µg/mL PHE (2 mM *D*‐penicillamine, 1 mM hypotaurine, and 245 µM epinephrine), 5.5 IU/mL heparin, and 6 mg/mL BSA. Each sperm batch was processed on a Percoll gradient (45% and 90%) to obtain sperm cells at a concentration of 1 × 10^6^ sperm/mL. Three different batches from three different bulls were used. Presumptive embryos were completely denuded using a pipette and were cultured in synthetic oviductal fluid with amino acids, sodium citrate, and inositol (SOF) supplemented with 5 mg/mL BSA, 22 µg/mL sodium pyruvate, 50 µg/mL gentamicin, and 2.5% FBS. The first cleavage was evaluated at 28–30 hpi (hours post‐insemination). The cleavage rate was assessed at 96 hpi of development, and blastocyst rate was evaluated at 168 hpi. Blastocysts were also collected to investigate the number of cells and evaluated regarding the stage of development in initial, typical blastocyst, expanded blastocyst, and hatched. Incubator conditions for embryo production were 38.5°C during all steps under 5% CO_2_ and air (20% oxygen) with saturating humidity. A total of three replicates of embryo production were performed.

### 2.12 Assessment of Blastocyst Cell Number

Blastocysts were collected and fixed for the investigation of the number of cells. For that, 22 blastocysts from control group and 29 blastocysts from the epEVs group were fixed for 15 min in paraformaldehyde 4% in PBS with 0.1% PVP. Next, blastocysts were permeabilized with 1% Triton X‐100 for 30 min. Finally, the embryos were stained with 10 µg/mL Hoechst for 15 min. Blastocyst slides were mounted with Prolong Antifade (Thermo Fisher Scientific, USA) and analyzed in Thunder Imager 3D Assay (Leica, Germany) fluorescence microscopy using the following settings: brightfield 550 nm, 50 ms and Hoechst LED405, 450 nm, 10 ms, under 400× magnification. The images were captured and analyzed using ImageJ software (NIH; https://imagej.net/ij/).

### 2.13 Statistical Analysis

Data regarding concentration of epEVs/sperm and period of incubation were analyzed with mixed models considering two class factors (concentration and time) and interaction using the donor of sperm as a random effect. ANOVA (analysis of variance) was performed using mixed models for the other experiments except to rates related to the embryo in vitro production that were compared using chi‐squared test. For miRNA expression (qPCR data), the delta‐Cq values were compared by ANOVA, while the results were plotted as the 2^−∆Cq^ values. Except for chi‐squared test, all the variables were tested regarding normality using Shapiro–Wilk test. When necessary, data were transformed, or outliers were removed. A minimal significance level of 5% (*p* ≤ 0.05) was adopted for all tests.

## 3 Results

The aim here was to investigate the effects of in vitro incubation of cauda epididymal sperm with cauda‐derived epEVs on sperm fertility potential. By using an in vitro model to demonstrate this effect, we aimed to establish a protocol for inducing a fast and dynamic modification of sperm fertility potential based on sperm interaction with epEVs. For that, we first obtained and characterized a pool of bovine epEVs. After that, we validated an in vitro model protocol to promote in vitro interaction between epEVs and sperm. Following this validation, the effect of this interaction was tested by showing molecular changes and using sperm for in vitro embryo production.

### 3.1 Isolation and Characterization of epEVs

**FIGURE 1:**
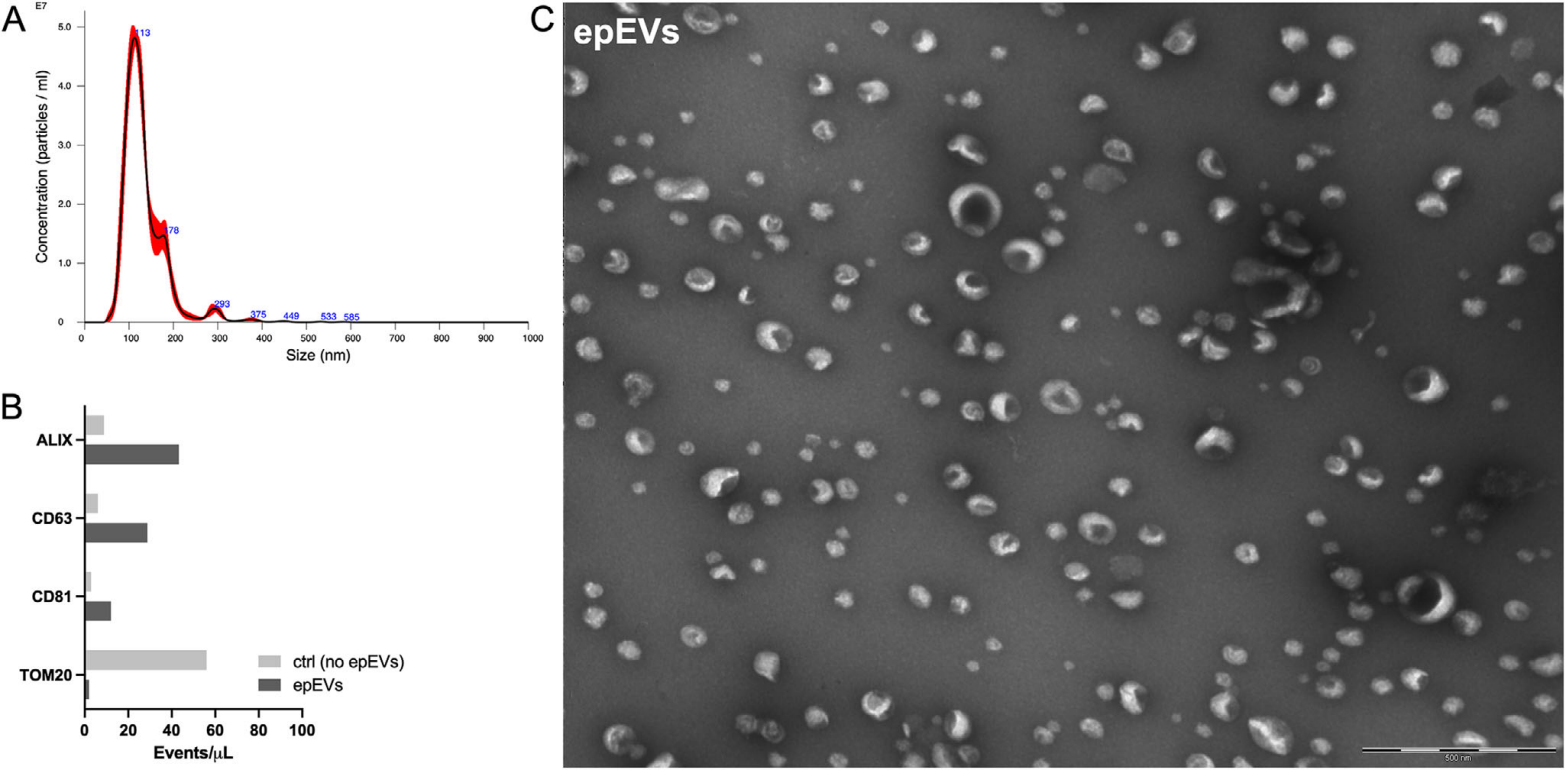
Characterization of epididymosomes (epEVs) isolated from bovine epididymal cauda. (A) Concentration and diameter sizes of the assessed nanoparticles. (B) Events per µL of the specific markers of extracellular vesicles (ALIX, CD63, and CD81) and negative marker (TOM20) in epEVs and in control (Ctrl; PBS with marker and without epEVs). (C) Transmission electron microscopy image of epEVs. Scale bar: 500 nm.

First, epEVs of cauda epididymal fluid were isolated and collected from five different bulls to form a pool. After that, we analyzed the pool of epEVs regarding size and concentration that resulted in 114.20 ± 3.60 nm of diameter and 3.48 × 10^9^ ± 1.84 × 10^8^ particles/mL of concentration (Figure 1A). We also confirmed the presence of epEVs’ specific markers ALIX, CD‐81, and CD‐63 by flow cytometry in the epEVs (Figure 1B). Regarding TOM20, a negative marker of EVs, epEVs had fewer positive events/µL compared to the control (Figure 1B). To demonstrate the antibody labeling efficiency, sperm were lysed and labeled with TOM20 antibody, and the data were shown by fluorescence intensity (positive control) in Table S1. Finally, the cup‐shaped epEVs morphology was confirmed by TEM (Figure 1C).

### 3.2 epEVs–Sperm Interaction Relies on epEVs: Sperm Ratio and Period of Incubation

**FIGURE 2:**
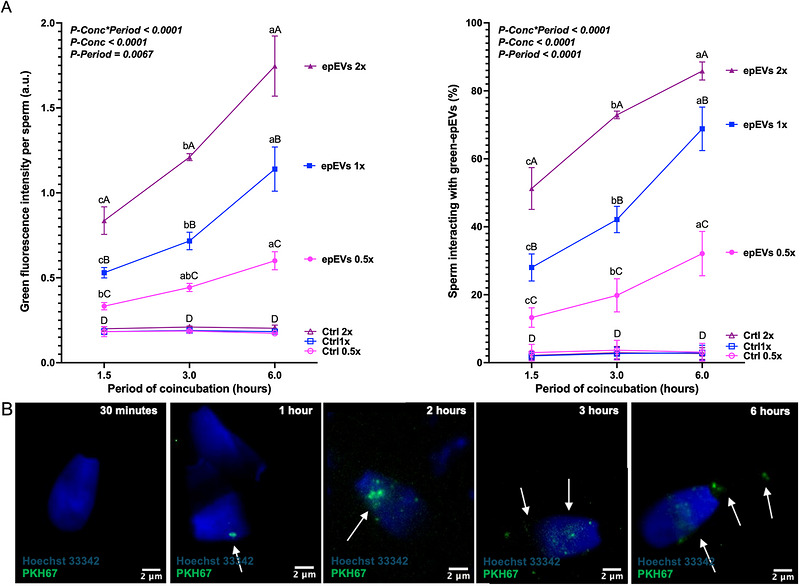
Interaction between epididymosomes (epEVs) and sperm during several concentrations and periods of interaction. (A) Mean and SEM of green fluorescent intensity per sperm (a.u.) and sperm interacting with green EVs (%) following treatments with concentrations of 500 (0.5×), 1000 (1×), and 2000 (2×) epEVs/sperm (epEVs 0.5×, epEVs 1×, and epEVs 2×) and respective controls (Ctrl 0.5×, Ctrl 1×, and Ctrl 2×) in different periods of incubation (1.5, 3, and 6 h). a.u., arbitrary units. Capital and lowercase letters indicate statistical difference between concentration and period, respectively. *p*‐values of concentration (Conc), period (Period), and interaction (Conc*Period) are presented in the graphics. A total of 5000 events positive to Hoechst were analyzed. The gates of flow cytometry analysis are represented in Figure S3. (B) Representative fluorescent microscopy images acquired on Axioplan 2 (Carl Zeiss, Germany) of sperm that interacted (epEVs) or not (Ctrl) with 1000 epEVs/sperm (1×) for 30 min, 1 h, 2 h, 3 h, and 6 h. Scale bar: 2 µm. The arrows indicate the presence of green epEVs interacting with sperm. PKH67 (green marker) was used to label the epEVs.

Since the protocol to promote in vitro interaction between epEVs and sperm is not established, we investigated the efficiency of sperm–epEVs interaction using different periods of incubation and concentrations of epEVs/sperm. Following epEVs characterization, epEVs were stained with a green marker (PKH67) and incubated with selected epididymal sperm in the proportions of 500 epEVs/sperm, 1000 epEVs/sperm, and 2000 epEVs/sperm. We also tested three periods of incubation: 1.5, 3, and 6 h. We found that there is an interaction between concentration of epEVs and the period of interaction for green fluorescence intensity per sperm (*p* < 0.0001) and sperm interacting with green epEVs percentage (*p* < 0.0001) (Figure 2A). In that regard, green fluorescence intensity per sperm displayed similar values for controls (0.18 ± 0.03 arbitrary units [a.u.], 0.18 ± 0.01 a.u., and 0.20 ± 0.01 a.u. for respectively 500, 1000, and 2000 epEVs/sperm incubated during 1.5 h; 0.18 ± 0.007 a.u., 0.19 ± 0.006 a.u., and 0.21 ± 0.006 a.u. for respectively 500, 1000, and 2000 epEVs/sperm incubated during 3 h; and 0.17 ± 0.003 a.u., 0.18 ± 0.003 a.u., 0.20 ± 0.01 a.u. for respectively 500, 1000, and 2000 epEVs/sperm incubated during 6 h) and higher values, according to Figure 2A, for epEVs (0.33 ± 0.02 a.u., 0.53 ± 0.03 a.u., and 0.83 ± 0.08 a.u. for respectively 500, 1000, and 2000 epEVs/sperm incubated during 1.5 h; 0.44 ± 0.02 a.u., 0.71 ± 0.05 a.u., and 1.21 ± 0.02 a.u. for respectively 500, 1000, and 2000 epEVs/sperm incubated during 3 h; 0.60 ± 0.05 a.u., 1.14 ± 0.13 a.u., and 1.74 ± 0.17 a.u. for respectively 500, 1000, and 2000 epEVs/sperm incubated during 6 h). On the same way, the percentage of sperm interacting with green epEVs displayed similar percentages for controls (2.96% ± 2.41%, 2.00% ± 1.25%, and 2.15% ± 1.18% for respectively 500, 1000, and 2000 epEVs/sperm incubated during 1.5 h; 3.69% ± 2.91%, 2.69% ± 1.83%, and 2.93% ± 1.85% for respectively 500, 1000, and 2000 epEVs/sperm incubated during 3 h; 3.09% ± 2.60%, 2.92% ± 2.23%, and 2.68% ± 1.71% for respectively 500, 1000, and 2000 epEVs/sperm incubated during 6 h) and higher percentages, according to Figure 2A, for epEVs (13.28% ± 2.85%, 28.01% ± 3.98%, and 51.26% ± 6.13% for respectively 500, 1000, and 2000 epEVs/sperm incubated during 1.5 h; 19.82% ± 4.88%, 42.13% ± 3.87%, and 72.93% ± 1.13% for respectively 500, 1000, and 2000 epEVs/sperm incubated during 3 h; 32.09% ± 6.54%, 68.82% ± 6.40%, and 85.84% ± 2.64% for respectively 500, 1000, and 2000 epEVs/sperm incubated during 6 h). In that regard, by increasing the concentration and period of interaction, epEVs interact at a higher rate with sperm as shown in Figure 2. Interestingly, the interaction between epEVs and sperm seems to gradually be more intensive according to the period of incubation (Figure 2B). Time‐lapse analysis revealed that this interaction persists even in motile sperm (Videos S2 and S3). In addition, the interaction between epEVs and sperm appears to be a tightly regulated process rather than a random event (Video S4). Altogether, these observations suggest that the interaction between sperm and epEVs is both nonrandom and non‐transient.

### 3.3 epEVs–Sperm Interaction at a Ratio of 1000 epEVs/Sperm and 3 h of Incubation Resulted in Modulation of Sperm Fertility Potential

**FIGURE 3:**
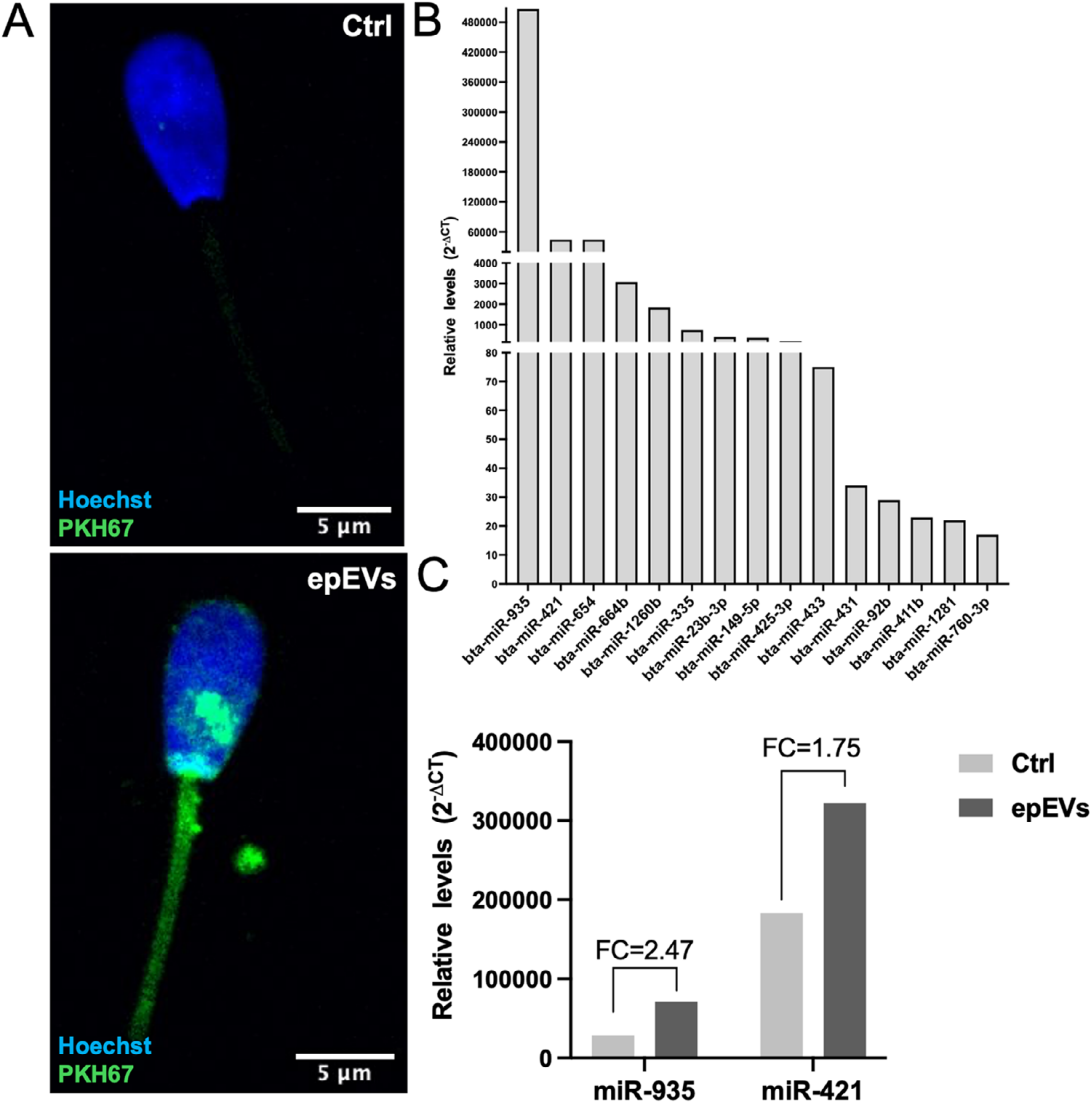
A concentration of 1000 epididymosomes (epEVs) per sperm and 3 h of incubation was selected as a fast and dynamic protocol to promote in vitro interaction between epEVs and sperm. (A) Representative confocal fluorescent microscopy images acquired on Mica Widefocal Live Cell (Leica, Germany) of sperm that interacted (epEVs) or not (Ctrl) with 1000 epEVs/sperm for 3 h. Scale bar: 5 µm. PKH67 (green marker) was used to label the epEVs. (B) Relative levels of the top 15 microRNAs detected in epEVs. The list of all detected microRNAs in epEVs are included as Tables S2 and S3. (C) Relative levels of the two out of five microRNAs investigated in sperm that interacted with epEVs. FC, fold‐change. Relative levels are shown as 2^−∆Cq^.

Since the interaction of sperm with epEVs depended on time and epEV abundance;  in the literature, most of the publications use 3 h of sperm–EVs coincubation [27]; and  in vivo, the proportion of epEVs/sperm is estimated at around 80 epEVs/sperm according to previous data from our group (Table S4), we decided to follow for the next experiment with the intermediate time and concentration, that is, 3 h of coincubation and 1000 epEVs/sperm (Figure 3A). Before the assessment of sperm fertility potential, we investigated if the interaction between sperm and epEVs promoted sperm epigenome changes. For that, we investigated target miRNAs in sperm following incubation with epEVs. We first identified the miRNAs that showed higher abundance in the pool of epEVs. This experiment showed that 49 miRNAs out of 380 assessed were detected in epEVs (bta‐miR‐935, ‐421, ‐654, ‐664b, ‐1260b, ‐335, ‐23b‐3p, ‐149‐5p, ‐425‐3p, ‐433, ‐431, ‐92b, ‐411b, ‐1281, ‐760‐3p, ‐1247‐3p, ‐568, ‐296‐5p, ‐450a, ‐432, ‐219, ‐340, ‐542‐5p, ‐658, ‐342, ‐362‐5p, ‐665, ‐1246, ‐197, ‐27a‐5p, ‐758, ‐1224, ‐223, ‐182, ‐378b, ‐376b, ‐375, ‐424‐3p, ‐761, let‐7b, ‐574, ‐let‐7e, ‐21‐3p, ‐1179, ‐940, ‐505, ‐339a, ‐455‐5p, and ‐129‐5p) (Figure 3B and Table S3). We then selected the top five miRNAs with higher abundance in epEVs (bta‐miR‐935, ‐421, ‐654, ‐664b, and ‐1260b) to verify the level of these miRNAs in sperm after incubation with epEVs. Interestingly, we found that two (miR‐935 and ‐421) out of five miRNAs were detected in sperm that interacted with epEVs with a fold‐change respectively of 2.47 and 1.75 (Figure 3C). Functional enrichment analysis indicated that the target transcripts of both miRNAs are involved in autophagy, oxytocin signaling, proteoglycans, and cellular senescence pathways (File S1).

**FIGURE 4:**
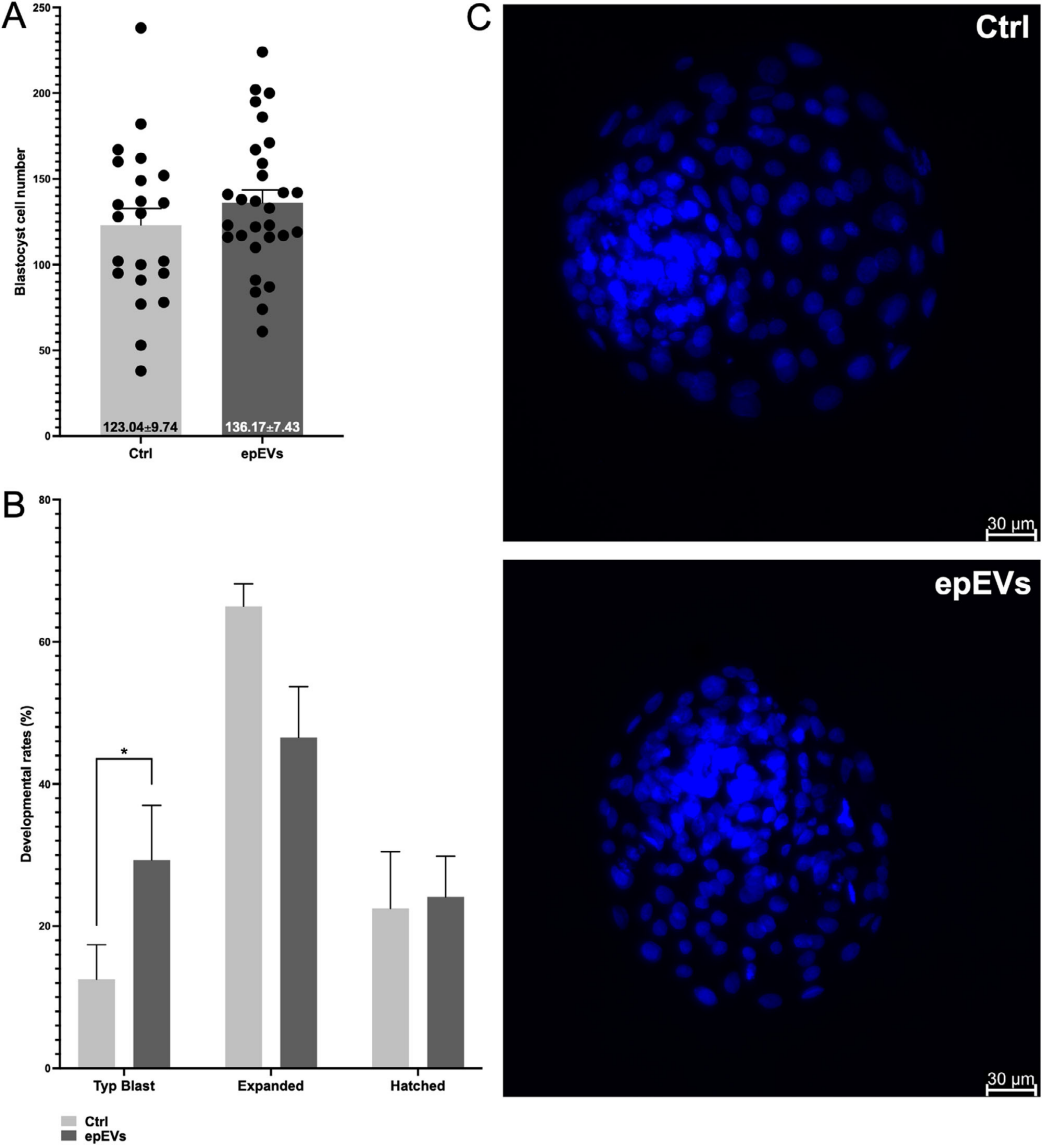
Quality of blastocysts produced with sperm that interacted with 1000 epididymosomes (epEVs)/sperm and 3 h of incubation before fertilization. (A) Mean and SEM of the number of cells counted in blastocysts collected from epEVs and control groups. Each dot on the graphics represents a blastocyst. (B) Mean and SEM of developmental rates of typical (Typ), expanded, and hatched blastocyst stages. (C) Representative confocal microscopy images of blastocysts stained with Hoechst from epEVs and control groups. Scale bar: 30 µm. Blastocyst of epEVs group were produced with sperm that interacted with 1000 epEVs/sperm for 3 h. Control group (Ctrl) was performed with sperm incubated at the same conditions without epEVs.

Once we established the protocol of incubation that promoted interaction and modulation of molecules in sperm, we coincubated frozen‐thawed epididymal sperm with epEVs immediately before IVF. After that, we used sperm to fertilize presumably mature oocytes. Regarding developmental rates, the first cleavage was similar (*p* = 0.48) between epEVs‐treated sperm and control sperm. On the other hand, although a similar cleavage rate was observed, it displayed a trend (*p* = 0.08) to be higher in epEVs‐treated sperm group. In addition, sperm treated with epEVs resulted in a higher (*p* = 0.04) blastocyst rate compared to control sperm (Table 1). Regarding quality of blastocysts, a similar number of cells was found between the groups, which was associated with a similar embryonic stage of expanded (*p* = 0.10; epEVs = 46.55% ± 7.13% [27/58]; control [ctrl] = 65.00% ± 3.17% [26/40]) and hatched (*p* = 0.77; epEVs = 24.13% ± 5.71% [14/58]; ctrl = 22.50% ± 7.96% [9/40]) blastocysts (Figure 4). Rate of typical blastocyst stage was higher (*p* = 0.05) in the epEVs group (29.31% ± 7.68% [17/58]) compared to the control (12.50% ± 4.89% [5/40]).

**TABLE 1:** Developmental rates of embryos produced with sperm that interacted with 1000 epididymosomes (epEVs)/sperm and 3 h of incubation before fertilization.

Sperm treatment	First cleavage rate^a^			Cleavage rate^b^		Blastocyst rate^c^	
	Oocytes	*N*	Mean ± SEM	*N*	Mean ± SEM	*N*	Mean ± SEM
Control	145	71	49.0 ± 8.2	88	60.7 ± 8.0	40	26.6 ± 5.6^b^
epEVs	149	80	53.7 ± 12.2	105	70.5 ± 13.8	58	38.9 ± 7.3^a^

Rates of first cleavage, cleavage, and blastocyst evaluated at 28 h post‐insemination (hpi), 96 hpi, and 168 hpi, respectively were evaluated. epEVs group was performed using post‐thawed epididymal sperm that interacted with 1000 epEVs/sperm for 3 h. Control group was performed with sperm incubated at the same conditions without epEVs. *N* represents the number of structures. Different letters in the same column indicate significant difference (*p* = 0.04).

^a^ Evaluated 28 hpi (hours post‐insemination).

^b^ Evaluated 96 hpi.

^c^Evaluated 168 hpi.

## 4 Discussion

**FIGURE 5:**
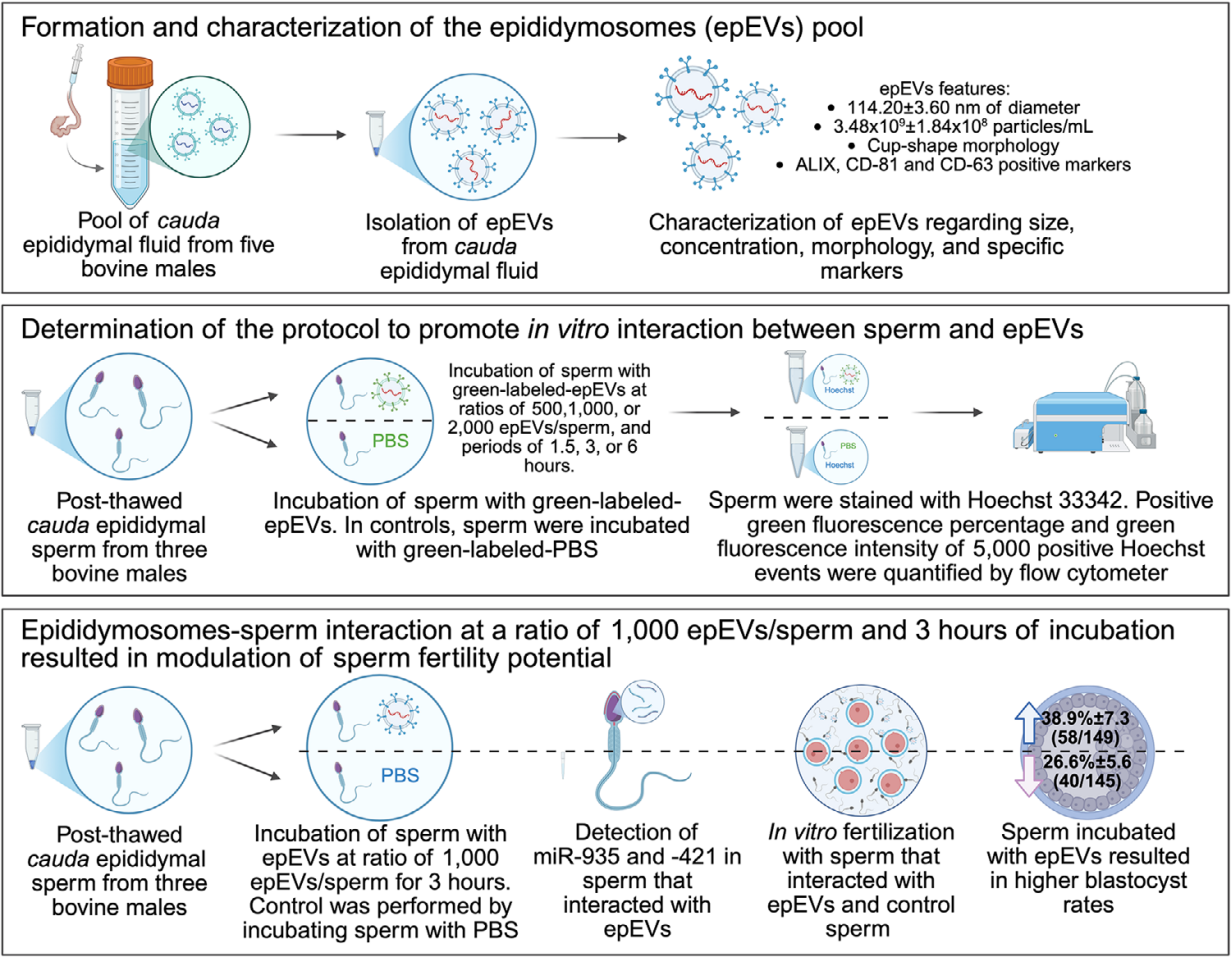
Graphical abstract figure summarizing methodology and main results. The figure shows first the methodology to obtain the pool of epididymosomes (epEVs) isolated from epididymal cauda of five bovine males. The pool of epEVs was characterized regarding size, concentration, morphology, and specific markers. Afterward, the protocol to promote in vitro interaction between sperm and epEVs was determined by incubating post‐thawed sperm obtained from epididymal cauda of three bovine males with epEVs at ratios of 500, 1000, or 2000 epEVs/sperm for periods of 1.5, 3, or 6 h. After incubation with epEVs, sperm were stained with Hoechst 33342 and analyzed on flow cytometer by detecting the percentage of sperm with positive green fluorescence and the green fluorescence intensity of sperm. For the analysis, sperm were considered as positive Hoechst events. Finally, the figure summarizes the main result of the experiment in the third panel: Sperm incubated with epEVs at ratio of 1000 epEVs/sperm for 3 h resulted in detection of miR‐935 and ‐421 in sperm, and the interaction between sperm and epEVs promoted changes in sperm fertility potential in which sperm that interacted with epEVs triggered higher blastocyst rates.

Herein, the aim was first to establish a protocol to promote in vitro interaction between sperm and epEVs to investigate the effects of the interaction on sperm fertilization potential. Briefly, the results revealed that it is feasible to modulate the fertilization potential of cryopreserved sperm using a fast approach that modulates the ability of sperm to fertilize and contribute to development, immediately before fertilization, by using epEVs (Figure 5). In addition to providing insights into modulation of male fertility for improving the production of embryos in assisted reproductive technology, these findings open avenues for shedding light on new therapies for male infertility.

Once morphologically formed in testis, sperm need to pass through the epididymis to acquire the ability to move and fertilize the oocyte in a process known as sperm maturation [5], in which sperm change their molecular profile along transit in epididymal segments [4]. Since sperm are considered as transcriptionally inert cells, these changes are mainly promoted by sperm interaction with epididymal epithelium [13]. These interactions are mainly mediated by small EVs, known as exosomes [12], released by epithelial cells from epididymis (i.e., epEVs) during transit through epididymal caput and corpus segments [5, 14]. In epididymal cauda, sperm are stored in a quiescent state [28, 29] since they already display the ability to move and fertilize the oocyte [5]. On the other hand, a dynamic modulation of epEVs from this segment is observed once isolated epEVs from epididymal cauda (called here as epEVs) exhibited a different molecular profile compared to epEVs collected from other epididymal segments [15, 27], and different subpopulations of cauda epEVs presented a distinct profile of proteins [30]. Thus, even though sperm are stored in a quiescent state, the microenvironment of epididymal cauda lumen is not static, and sperm may modulate their molecular profile during the period of storage that corresponds to more than 50% of the period that sperm remain in epididymis.

Considering that sperm may modulate their molecular content during storage, we hypothesized that interaction of post‐thawed sperm with epEVs immediately before fertilization could modulate sperm fertility ability. In that regard, herein, we aimed to investigate it by means of an in vitro assay. For this, epEVs from epididymal cauda fluid (epEVs) were isolated by retrograde perfusion, adapting the technique described in Belleannée et al. [15]. To certify that this method of epididymal fluid obtention was free from contamination of epididymal epithelium or blood, at the beginning of the technique validation, we stained smears with the collected epididymal fluid to check the presence of somatic cells, which was minimal (< 0.5%) in the samples. Thus, we followed this method to obtain the samples to develop this study. Once obtained, epididymal fluids were processed to isolate epEVs by ultracentrifugation, a method well established for obtention of epEVs [11]. After that, epEVs were characterized regarding size, morphology, and specific markers. The diameter of the isolated epEVs was around 100 nm, in accordance with previous studies that isolated epEVs [11]. The morphology and specific markers were found in accordance with the guidelines established by the characterization of exosomes [31].

Confirming the cup‐shaped morphology, the homogeneity size between epEVs population, the presence of specific markers ALIX, CD‐63, and CD‐81, and the absence of contaminants, using as a control a marker of mitochondria organelles, the isolated epEVs were stained with the green‐marker PKH67, that intercalates into the lipid bilayer of membranes.   Since the green marker PKH67 stains membranes, staining of epEVs was followed, by neutralization of the dye in accordance with Da Silveira et al. [32], and the samples of epEVs were washed. After that, we isolated the green epEVs by ultracentrifugation and used them to incubate with sperm. This protocol was also performed with PBS prior to using it for the negative control groups. Thus, the PBS was previously “stained” with PKH67. In that regard, we considered that a remaining green marker during the staining and isolation of epEVs could be mimicked by these control conditions. A similar protocol was performed by Da Silveira et al. [32] and Lange‐Consiglio et al. [33].

Regarding the quantity of epEVs, as we did not find a precise information in previous studies, we decided to estimate the physiological concentrations in the epididymis and use this information to decide the quantity of epEVs to use in our study. In that regard, the ratio of epEVs/sperm was based on previous data of our group, in which we estimated that the in vivo proportion of epEVs per sperm in bovine epididymis cauda is around 81.38 ± 15.26 epEVs/sperm (Table S4). It is important to highlight that this ratio was calculated based on a small population (six animals) and that this could vary between species, age, and other parameters. However, to determine the ratios to be tested here, we considered that the diameter of the epididymal duct is extremely narrow and the probability of an in vivo dynamic interaction between epEVs and sperm is higher than in vitro. Thus, we tested higher proportions (∼6×, 12×, and 24× more, respectively; 500, 1000, and 2000 epEVs/sperm) of epEVs per sperm to increase the chance of interaction of sperm with epEVs in a static model. Regarding the period of incubation, we considered a previous study in which interaction was provided for 3 h with a mouse model [27]. Thus, we tested half of this period and the double of that period. In the end, we found that sperm interact with epEVs in accordance with the quantity of epEVs and the period of incubation. Interestingly, Lange‐Consiglio et al. [33] studying the interaction of bovine EVs from seminal plasma and sperm from ejaculate tested 1, 2, 3, 4, 5, and 6 h of incubation and noted that 1 h of incubation was sufficient to promote interaction with a 400 × 10^6^ EVs/mL. In our study, we determined the epEVs concentration ratio based on sperm, but we estimated that we added around 1 × 10^9^ epEVs/mL when adding 1000 epEVs/sperm. In our study, we did not find a plateau of interaction, even though we consider that there is one.

Since we found that the interaction increased according to epEVs concentration and period of incubation, the period of 3 h that was previously used by Reilly et al. [27] was used in this study. In accordance with the period, the concentration of epEVs was the intermediate one as well. Thus, we added around 10 times more epEVs per sperm compared to the estimation that we performed on in vivo cauda microenvironment of bulls. Using this protocol, we detected that epEVs interacted with post‐acrosomal region and intermediate piece with more intensity compared to acrosomal region. In fact, previous studies have detected that related receptors of epEVs are mainly localized in post‐acrosomal region of sperm head [34]. In addition, Lange‐Consiglio et al. [33] found that EVs from seminal plasma interact with more intensity with intermediate piece of sperm. Also, we found that the intensity of the interaction with sperm head and tail seems to increase with the increasing period of incubation. However, in this study, we did not quantify it. Thus, it is necessary for more studies to provide information on the pattern of epEVs interaction with sperm. Interestingly, time‐lapse assessment conducted in this study indicates that the interaction between sperm and epEVs is maintained during sperm motility (Video S3). An additional key observation supported by time‐lapse analysis is that the interaction between sperm and epEVs appears to be nonrandom (Video S4).

Following confirmation of interaction between the marked epEVs and sperm, the protocol of interaction using 1000 epEVs per sperm and 3 h of incubation was used to verify molecular changes promoted by epEVs in sperm. For that, first, we established the miRNA profile of the epEVs used in the study. Interestingly, 49 out of 380 miRNAs were detected in the isolated epEVs, and from these 49 miRNAs, Belleannée et al. [15] found that miR‐654 and miR‐1224 were detected higher in bovine cauda epEVs compared to caput epEVs. In addition, Reilly et al. [27] found that miR‐421, ‐664‐3p, ‐335, ‐23b‐3p, and ‐425 are presented in cauda epEVs from mice model. All these miRNAs were detected in high levels in epEVs. In our study, from the 49 detected in epEVs, the top five were investigated in sperm that interacted with epEVs. Interestingly, two out of the five miRNAs (miR‐935 and ‐421) were detected in sperm that interacted with epEVs, indicating that probably epEVs were delivering these molecules to sperm. Regarding miR‐935, it was already detected with a decreased level in sperm from males with a higher number of morphological defects [35]. On the other hand, miR‐421 was associated with acrosomal reaction and mitochondrial function according to Kasimanickam and Kasimanickam [36]. In addition, pathways of autophagy, oxytocin signaling, proteoglycans, and cellular senescence are related with the target transcripts of miR‐935 and ‐421. Since epEVs may increase these miRNAs in sperm and these pathways are important to epididymis function, we speculate that these epEVs deliver key molecules to sperm to promote epididymal functions of sperm selection and sperm release. However, epEVs carry other molecules and could deliver proteins, lipids, and other molecules of RNAs as circRNAs to sperm [37, 38, 39]. These other molecules delivered by epEVs to sperm could also impact sperm fertility ability. Thus, we need to consider that other components could also change the molecular profile of sperm that were not investigated in this study. Additionally, considering that sperm from the epididymal cauda were previously exposed to epEVs in vivo, it should be noted that the changes in sperm induced by epEVs observed in this study may be related to sperm overexposure to epEVs, and/or to nonphysiological conditions, since sperm are usually ejaculated with epEVs and this interaction may have been prematurely interrupted.

Since sperm are transcriptionally inert and not able to translate proteins as well, the main impact of receiving molecules by interacting with epEVs is on fertilization ability by paternal contribution to development. Recently, sperm were recognized as cells that, even though presenting a diminished size, can contribute to development with molecules as proteins and miRNAs. In that regard, sperm deliver PCL‐Ζ protein that activates the oocyte [40], and miRNAs as miR‐34c [17], miR‐191 [18], miR‐449b [16], and miR‐216b [41], that impact sperm fertility ability and embryonic development in mice, humans, and bovines. Thus, the main role of interacting with epEVs is probably to promote modulation of sperm fertility potential, which includes verifying the paternal effect during embryonic development. Herein, we used the sperm that interacted with epEVs to produce embryos and verify the effect on sperm ability to fertilize and contribute to development. Using the in vitro embryo production model is highly appropriate for this approach, since sperm are not challenged to reach the fertilization site. In that regard, in a previous study of our group, we detected difference in fertility by impairment of development using the in vitro model, that the in vivo model was not able to detect [41]. Regarding fertilization, the first cleavage did not signal a difference in fertilization rates in the present study. However, the blastocyst rate was higher from sperm that interacted with epEVs, which raised questions regarding the key contributors of male fertility potential. Regarding the miRNAs found in the study, only miR‐935 was associated with fertility. Interestingly, this miRNA was not found in bovine oocytes and zygotes in the study of Paulson et al. [42]. On the other hand, miR‐421 was detected in oocytes and seems to increase the levels in zygotes and two‐cell embryos [42].

In conclusion, regarding sperm's ability to accurately reach the fertilization site and fertilize the oocyte, the sperm features of motility, morphology, high potential of mitochondrial membranes, and integrity of plasma and acrosome membranes, as well as of DNA, are well established, with numerous techniques to assess them (reviewed by Alves et al.) [2]. On the other hand, the specific molecules signature of sperm that are involved in the mechanism of sperm contribution to development is also important to fulfill sperm fertility potential; however, it remains poorly investigated, and the mechanisms to manipulate it are not clearly shown [17]. In addition to the technical challenges in precisely detecting sperm molecular cargo, sperm are considered transcriptionally and translationally inert cells [43, 44]. Even part of sperm molecular cargo is acquired during formation of sperm; interaction with somatic cells represents a major mechanism of modulation of molecular profile [45, 46, 47]. This complex network of interactions makes it challenging to determine which molecules are key for male fertility and which specific interactions are required to modify the sperm molecular cargo. However, since sperm remains in epididymis at least 5–10 days before fertilization and is stored until the moment of ejaculation in this organ, it is expected that factors that interfere in epididymis could modify the interaction of sperm with epididymis and, hence, fertilization and paternal contribution. Since epEVs are the main mechanism of communication and sperm remain in epididymal cauda stored for a significant period, we demonstrated here that the interaction of sperm with cauda epEVs changed sperm ability to contribute to embryo development. These findings provide valuable information regarding male contribution to development and shed light on new therapies for male infertility and fast modulation of sperm fertility potential. Also, these outcomes demonstrate that humans and animals that pass through a stress or a condition that change the cauda epEVs profile, may present disturbances in male fertility immediately before fertilization.

## Author Contributions

M.B.R.A. conceived the study, designed and performed the experiments, prepared data presentation, analyzed and interpreted the data, and wrote the manuscript. M.A.A., A.B.B.M., L.G.H., and R.B.R. performed the experiments and interpreted the data. F.V.M. and J.C.S. provided resources and discussed methodology and data. F.P. conceived the study, provided resources, oversaw the research planning and execution, analyzed the data, and wrote the manuscript. All authors reviewed the manuscript.

## Funding

This research was funded by the Sao Paulo Research Foundation (FAPESP) (grants 2015/21829‐9, 2019/23685‐5, 2021/08759‐2, 2021/09886‐8, 2022/01433‐7, 2022/01505‐8, 2024/05151‐1, 2024/10363‐8, and 2024/13164‐6) and the National Council for Scientific and Technological Development (CNPq) (grant 305977/2024‐5). This study was financed in part by the Coordenação de Aperfeiçoamento de Pessoal de Nível Superior—Brasil (CAPES)—finance code 001.

## Conflicts of Interest

The authors declare no conflicts of interest.

## Supporting information




**Supporting File 1**: andr70267‐sup‐0001‐videoS1.mov

**Supporting File 2**: andr70267‐sup‐0002‐videoS2.mov

**Supporting File 3**: andr70267‐sup‐0003‐videoS3.mov

**Supporting File 4**: andr70267‐sup‐0004‐videoS4.mov

**Supporting File 5**: andr70267‐sup‐0005‐SuppMat.xlsx

**Supporting File 6**: andr70267‐sup‐0006‐SuppMat.docx

## Data Availability

The data that support the findings of this study are available from the corresponding author upon reasonable request.

## References

[1] L. Boeri , H. Kandil , and J. Ramsay , “Idiopathic Male Infertility – What Are We Missing?,” Arab Journal of Urology 23, no. 3 (2025): 215–229, 10.1080/20905998.2024.2381972. 40747475 PMC12308862

[2] M. B. R. Alves , E. C. C. Celeghini , and C. Belleannée , “From Sperm Motility to Sperm‐Borne MicroRNA Signatures: New Approaches to Predict Male Fertility Potential,” Frontiers in Cell and Developmental Biology 8 (2020): 791, 10.3389/fcell.2020.00791. 32974342 PMC7471662

[3] S. Skerget , M. A. Rosenow , K. Petritis , and T. L. Karr , “Sperm Proteome Maturation in the Mouse Epididymis,” PLoS One 10, no. 11 (2015): e0140650, 10.1371/journal.pone.0140650. 26556802 PMC4640836

[4] B. Nixon , S. J. Stanger , B. P. Mihalas , et al., “The MicroRNA Signature of Mouse Spermatozoa Is Substantially Modified During Epididymal Maturation,” Biology of Reproduction 93, no. 4 (2015): 91, 10.1095/biolreprod.115.132209. 26333995

[5] B. Robaire and B. T. Hinton , “The Epididymis,” in Knobil and Neill's Physiology of Reproduction (Elsevier, 2015), 691–771, 10.1016/B978-0-12-397175-3.00017-X.

[6] M. G. Gervasi and P. E. Visconti , “Molecular Changes and Signaling Events Occurring in Spermatozoa During Epididymal Maturation,” Andrology 5, no. 2 (2017): 204–218, 10.1111/andr.12320. 28297559 PMC5354101

[7] J. L. Dacheux and F. Dacheux , “New Insights Into Epididymal Function in Relation to Sperm Maturation,” Reproduction 147, no. 2 (2014): R27–R42, 10.1530/REP-13-0420. 24218627

[8] T. G. Cooper , “Sperm Maturation in the Epididymis: A New Look at an Old Problem,” Asian Journal of Andrology 9, no. 4 (2007): 533–539, 10.1111/j.1745-7262.2007.00285.x. 17589792

[9] T. G. Păunesc , W. W. C. Shum , C. Huynh , et al., “High‐Resolution Helium Ion Microscopy of Epididymal Epithelial Cells and Their Interaction With Spermatozoa,” Molecular Human Reproduction 20, no. 10 (2014): 929–937, 10.1093/molehr/gau052. 25015675 PMC4172170

[10] M. A. Battistone , R. G. Spallanzani , A. C. Mendelsohn , et al., “Novel Role of Proton‐Secreting Epithelial Cells in Sperm Maturation and Mucosal Immunity,” Journal of Cell Science 133, no. 5 (2020): jcs233239, 10.1242/jcs.233239. PMC7003979 31636115

[11] G. Frenette , J. Girouard , and R. Sullivan , “Comparison Between Epididymosomes Collected in the Intraluminal Compartment of the Bovine Caput and Cauda Epididymidis,” Biology of Reproduction 75, no. 6 (2006): 885–890, 10.1095/biolreprod.106.054692. 16943362

[12] G. Raposo and W. Stoorvogel , “Extracellular Vesicles: Exosomes, Microvesicles, and Friends,” Journal of Cell Biology 200, no. 4 (2013): 373–383, 10.1083/jcb.201211138. 23420871 PMC3575529

[13] H. Chen , M. B. R. Alves , and C. Belleannée , “Contribution of Epididymal Epithelial Cell Functions to Sperm Epigenetic Changes and the Health of Progeny,” Human Reproduction Update 28, no. 1 (2021): 51–66, 10.1093/humupd/dmab029. 34618012

[14] R. Sullivan , G. Frenette , and J. Girouard , “Epididymosomes Are Involved in the Acquisition of New Sperm Proteins During Epididymal Transit,” Asian Journal of Andrology 9, no. 4 (2007): 483–491, 10.1111/j.1745-7262.2007.00281.x. 17589785

[15] C. Belleannée , É. Calvo , J. Caballero , and R. Sullivan , “Epididymosomes Convey Different Repertoires of MicroRNAs Throughout the Bovine Epididymis,” Biology of Reproduction 89, no. 2 (2013): 30, 10.1095/biolreprod.113.110486. 23803555

[16] M. Wang , Y. Gao , P. Qu , et al., “Sperm‐Borne miR‐449b Influences Cleavage, Epigenetic Reprogramming and Apoptosis of SCNT Embryos in Bovine,” Scientific Reports 7, no. 1 (2017): 13403, 10.1038/s41598-017-13899-8. 29042680 PMC5645405

[17] W. M. Liu , R. T. K. Pang , P. C. N. Chiu , et al., “Sperm‐Borne MicroRNA‐34c Is Required for the First Cleavage Division in Mouse,” Proceedings of the National Academy of Sciences 109, no. 2 (2012): 490–494, 10.1073/pnas.1110368109. PMC3258645 22203953

[18] H. Xu , X. Wang , Z. Wang , et al., “MicroRNA Expression Profile Analysis in Sperm Reveals hsa‐mir‐191 as an Auspicious Omen of In Vitro Fertilization,” BMC Genomics 21, no. 1 (2020): 165, 10.1186/s12864-020-6570-8. 32066367 PMC7027243

[19] F. Manfrevola , N. Mosca , V. G. Mele , et al., “Epididymal‐Born circRNA Cargo and Its Implications in Male Fertility,” International Journal of Molecular Sciences 26, no. 6 (2025): 2614, 10.3390/ijms26062614. 40141256 PMC11942175

[20] C. C. Conine , F. Sun , L. Song , J. A. Rivera‐Pérez , and O. J. Rando , “Small RNAs Gained During Epididymal Transit of Sperm Are Essential for Embryonic Development in Mice,” Developmental Cell 46, no. 4 (2018): 470–480.e3, 10.1016/j.devcel.2018.06.024. 30057276 PMC6103825

[21] M. A. de Almeida , L. G. Haupenthal , A. N. Silva , et al., “A Longer Period of Epididymal Sperm Interaction With Extender Components During Cryopreservation Improves Sperm Quality, Decreases the Size of Sperm Distal Cytoplasmic Droplets, and Changes the Number of Nanoparticles in the Extender,” Cryobiology 115 (2024): 104901, 10.1016/j.cryobiol.2024.104901. 38754687

[22] M. B. R. Alves , R. P. de Arruda , and L. Batissaco , “Changes in miRNA Levels of Sperm and Small Extracellular Vesicles of Seminal Plasma Are Associated With Transient Scrotal Heat Stress in Bulls,” Theriogenology 161 (2021): 26–40, 10.1016/j.theriogenology.2020.11.015. 33278692

[23] J. G. Ferst , M. A. Chaves , A. N. Silva , et al., “Uterine Extracellular Vesicles Can Emulate the Long‐Term Effects of Post‐Partum Negative Energy Balance in Dairy Cows,” Molecular Reproduction and Development 92, no. 10 (2025): e70062, 10.1002/mrd.70062. 41128104 PMC12548006

[24] S. A. Bustin , V. Benes , J. A. Garson , et al., “The MIQE Guidelines: Minimum Information for Publication of Quantitative Real‐Time PCR Experiments,” Clinical Chemistry 55, no. 4 (2009): 611–622, 10.1373/clinchem.2008.112797. 19246619

[25] T. D. Schmittgen and K. J. Livak , “Analyzing Real‐Time PCR Data by the Comparative CT Method,” Nature Protocols 3, no. 6 (2008): 1101–1108, 10.1038/nprot.2008.73. 18546601

[26] K. J. Livak and T. D. Schmittgen , “Analysis of Relative Gene Expression Data Using Real‐Time Quantitative PCR and the 2‐ΔΔCT Method,” Methods 25, no. 4 (2001): 402–408, 10.1006/meth.2001.1262. 11846609

[27] J. N. Reilly , E. A. McLaughlin , S. J. Stanger , et al., “Characterisation of Mouse Epididymosomes Reveals a Complex Profile of MicroRNAs and a Potential Mechanism for Modification of the Sperm Epigenome,” Scientific Reports 6, no. 1 (2016): 31794, 10.1038/srep31794. 27549865 PMC4994100

[28] S. Breton , P. J. S. Smith , B. Lui , and D. Brown , “Acidification of the Male Reproductive Tract by a Proton Pumping(H+)‐ATPase,” Nature Medicine 2, no. 4 (1996): 470–472, 10.1038/nm0496-470. 8597961

[29] T. S. Acott and D. W. Carr , “Inhibition of Bovine Spermatozoa by Caudal Epididymal Fluid: II. Interaction of pH and a Quiescence Factor,” Biology of Reproduction 30, no. 4 (1984): 926–935, 10.1095/biolreprod30.4.926. 6329337

[30] G. Frenette , J. Girouard , O. D'Amours , N. Allard , L. Tessier , and R. Sullivan , “Characterization of Two Distinct Populations of Epididymosomes Collected in the Intraluminal Compartment of the Bovine Cauda Epididymis,” Biology of Reproduction 83, no. 3 (2010): 473–480, 10.1095/biolreprod.109.082438. 20554923

[31] C. Théry , K. W. Witwer , E. Aikawa , et al., “Minimal Information for Studies of Extracellular Vesicles 2018 (MISEV2018): A Position Statement of the International Society for Extracellular Vesicles and Update of the MISEV2014 Guidelines,” Journal of Extracellular Vesicles 7, no. 1 (2018): 1535750, 10.1080/20013078.2018.1535750. 30637094 PMC6322352

[32] J. C. da Silveira , G. M. Andrade , M. del Collado , et al., “Supplementation With Small‐Extracellular Vesicles From Ovarian Follicular Fluid During In Vitro Production Modulates Bovine Embryo Development,” PLoS One 12, no. 6 (2017): e0179451, 10.1371/journal.pone.0179451. 28617821 PMC5472319

[33] A. Lange‐Consiglio , E. Capra , N. Monferini , et al., “Extracellular Vesicles From Seminal Plasma to Improve Fertilizing Capacity of Bulls,” Reproduction and Fertility 3, no. 4 (2022): 313–327, 10.1530/RAF-22-0037. 36374278 PMC9782411

[34] W. Zhou , S. J. Stanger , A. L. Anderson , et al., “Mechanisms of Tethering and Cargo Transfer During Epididymosome‐Sperm Interactions,” BMC Biology 17, no. 1 (2019): 35, 10.1186/s12915-019-0653-5. 30999907 PMC6474069

[35] A. Salas‐Huetos , J. Blanco , F. Vidal , et al., “Spermatozoa From Patients With Seminal Alterations Exhibit a Differential Micro‐Ribonucleic Acid Profile,” Fertility and Sterility 104, no. 3 (2015): 591–601, 10.1016/j.fertnstert.2015.06.015. 26143365

[36] V. Kasimanickam and R. Kasimanickam , “MicroRNAs and Their Associated Genes Regulating the Acrosome Reaction in Sperm of High‐ Versus Low‐Fertility Holstein Bulls,” Animals 14, no. 6 (2024): 833, 10.3390/ani14060833. 38539931 PMC10967381

[37] F. Barrachina , M. A. Battistone , J. Castillo , et al., “Sperm Acquire Epididymis‐Derived Proteins Through Epididymosomes,” Human Reproduction 37, no. 4 (2022): 651–668, 10.1093/humrep/deac015. 35137089 PMC8971652

[38] B. Nixon , G. N. De Iuliis , H. M. Hart , et al., “Proteomic Profiling of Mouse Epididymosomes Reveals Their Contributions to Post‐Testicular Sperm Maturation,” Molecular & Cellular Proteomics 18, no. Suppl 1 (2018): S91–S108, 10.1074/mcp.RA118.000946. 30213844 PMC6427233

[39] R. Sullivan and F. Saez , “Epididymosomes, Prostasomes, and Liposomes: Their Roles in Mammalian Male Reproductive Physiology,” Reproduction 146, no. 1 (2013): R21–R35, 10.1530/REP-13-0058. 23613619

[40] C. M. Saunders , M. G. Larman , J. Parrington , et al., “PLCζ: A Sperm‐Specific Trigger of Ca^2+^ Oscillations in Eggs and Embryo Development,” Development 129, no. 15 (2002): 3533–3544, 10.1242/dev.129.15.3533. 12117804

[41] M. B. R. Alves , R. P. de Arruda , T. H. C. De Bem , et al., “Sperm‐Borne miR‐216b Modulates Cell Proliferation During Early Embryo Development via K‐RAS,” Scientific Reports 9, no. 1 (2019): 10358, 10.1038/s41598-019-46775-8. 31316130 PMC6637201

[42] E. E. Paulson , E. L. Fishman , R. M. Schultz , and P. J. Ross , “Embryonic MicroRNAs Are Essential for Bovine Preimplantation Embryo Development,” PNAS 119, no. 45 (2022): e2212942119, 10.1073/pnas. 36322738 PMC9659414

[43] X. Ren , X. Chen , Z. Wang , and D. Wang , “Is Transcription in Sperm Stationary or Dynamic?,” Journal of Reproduction and Development 63, no. 5 (2017): 439–443, 10.1262/jrd.2016-093. 28845020 PMC5649092

[44] R. A. Lymbery , F. Garcia‐Gonzalez , and J. P. Evans , “Silent Cells? Potential for Context‐Dependent Gene Expression in Mature Sperm,” Proceedings of the Royal Society B: Biological Sciences 292, no. 2038 (2025): 20241516, 10.1098/rspb.2024.1516. PMC11706646 39772960

[45] P. H. Hung and S. S. Suarez , “Alterations to the Bull Sperm Surface Proteins That Bind Sperm to Oviductal Epithelium,” Biology of Reproduction 87, no. 4 (2012): 88, 10.1095/biolreprod.112.099721. 22837481 PMC4434996

[46] A. Boerke , S. J. Dieleman , and B. M. Gadella , “A Possible Role for Sperm RNA in Early Embryo Development,” Theriogenology 68 (2007): S147–S155, 10.1016/j.theriogenology.2007.05.058. 17583784

[47] A. P. B. de Souza , Â. M. Schorr‐Lenz , F. Lucca , and I. Cunha Bustamante‐Filho , “The Epididymis and Its Role on Sperm Quality and Male Fertility,” Animal Reproduction 14 (2017): 1234–1244, 10.21451/1984-3143-AR955.

