# Supplementary material for: In Vitro Sperm–Epididymosomes Interaction Immediately Before Fertilization Changes Sperm Fertility Potential
Source: Andrology. 2026 May 29;14(6):1757–70. doi: 10.1111/andr.70267 (PMC13432530; doi:10.1111/andr.70267)
Supplement: Supplementary file 6 — Supporting File 6: andr70267‐sup‐0006‐SuppMat.docx [file ANDR-14-1757-s006.docx]

**Title**

***In vitro* sperm-epididymosomes interaction immediately before fertilization changes sperm fertility potential**

**Authors**

Maíra Bianchi Rodrigues **Alves^1^***, Maria Alice de **Almeida^2^**, Ana Beatriz Bossois **Moura^2^**, Laura Gabrielli **Haupenthal^2^**, Raissa Braido **Rangel^2^**, Flávio Vieira **Meirelles^2^**, Juliano Coelho da **Silveira^2^**, Felipe **Perecin^2^**

**Authors’ institutions**

**¹Department of Pathology, Theriogenology and One Healthy, School of Agricultural and Veterinary Sciences, São Paulo State University, Jaboticabal, São Paulo, Brazil.**

**^2^Department of Veterinary Medicine, School of Animal Science and Food Engineering, University of São Paulo, Pirassununga, São Paulo, Brazil.**

**Authors’ email addresses**

**MBRA:** maira.bianchi@unesp.br

**MAA: maa.almeida@usp.br**

**ABBM:** ana.moura@usp.br

**LGH: laura.gabrielli@usp.br**

**RBR:** raissabraido@usp.br

**FVM: meirellf@usp.br**

**JCS: julianodasilveira@usp.br**

**FP: fperecin@usp.br**

**Short Title**

Sperm-epididymosomes **crosstalk changes male fertility**

**Keywords**

**Paternal contribution, exosomes, miRNAs, epididymis, IVF, embryo.**

***Corresponding author’s contact information**

**maira.bianchi@unesp.br**

**ORCID: https://orcid.org/0000-0002-9480-6608**

**Postal code: Professor Paulo Donato Castelane, 14884-900, Jaboticabal, SP, Brazil.**

**Supplementary Data**

**Supplementary Figure S1. Epididymal preparation for epididymis *cauda* retrograde perfusion.** In ***A***, both epididymis-testis complex of one bull. In the image is indicated the epididymal segments (*caput*, *corpus* and *cauda*) and testis. In ***B***, it is represented the procedure of separation of epididymis from testis. In ***C***, both epididymis of one bull after separation from testis is shown. In the image of the left is indicated the entire epididymis with all segments together. In the image of the right, epididymal segments (*caput*, *corpus* and *cauda*) were separated. For perfusion of epididymal *cauda,* it was preserved the deferent duct.

***
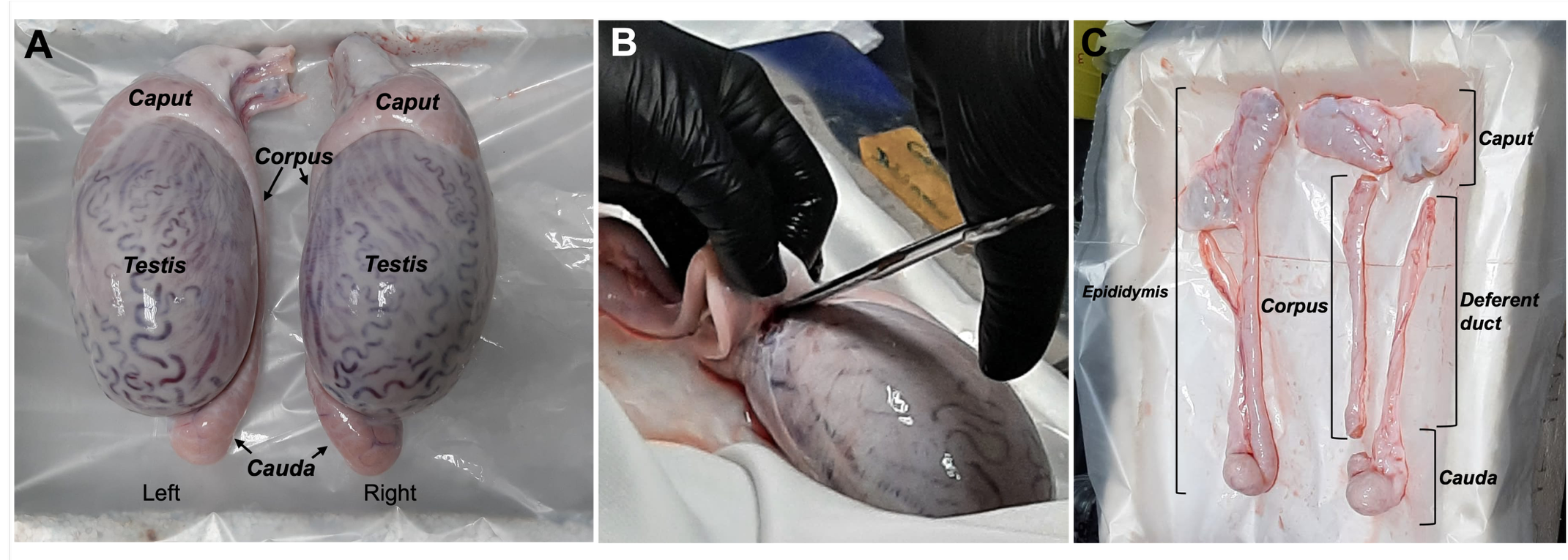
***

**Supplementary Figure S2. Retrograde perfusion of epididymal cauda.** A 22G catheter connected to a 10 mL syringe was introduced into the deferent duct and PBS was injected to perfuse the epididymal *cauda* duct.

**
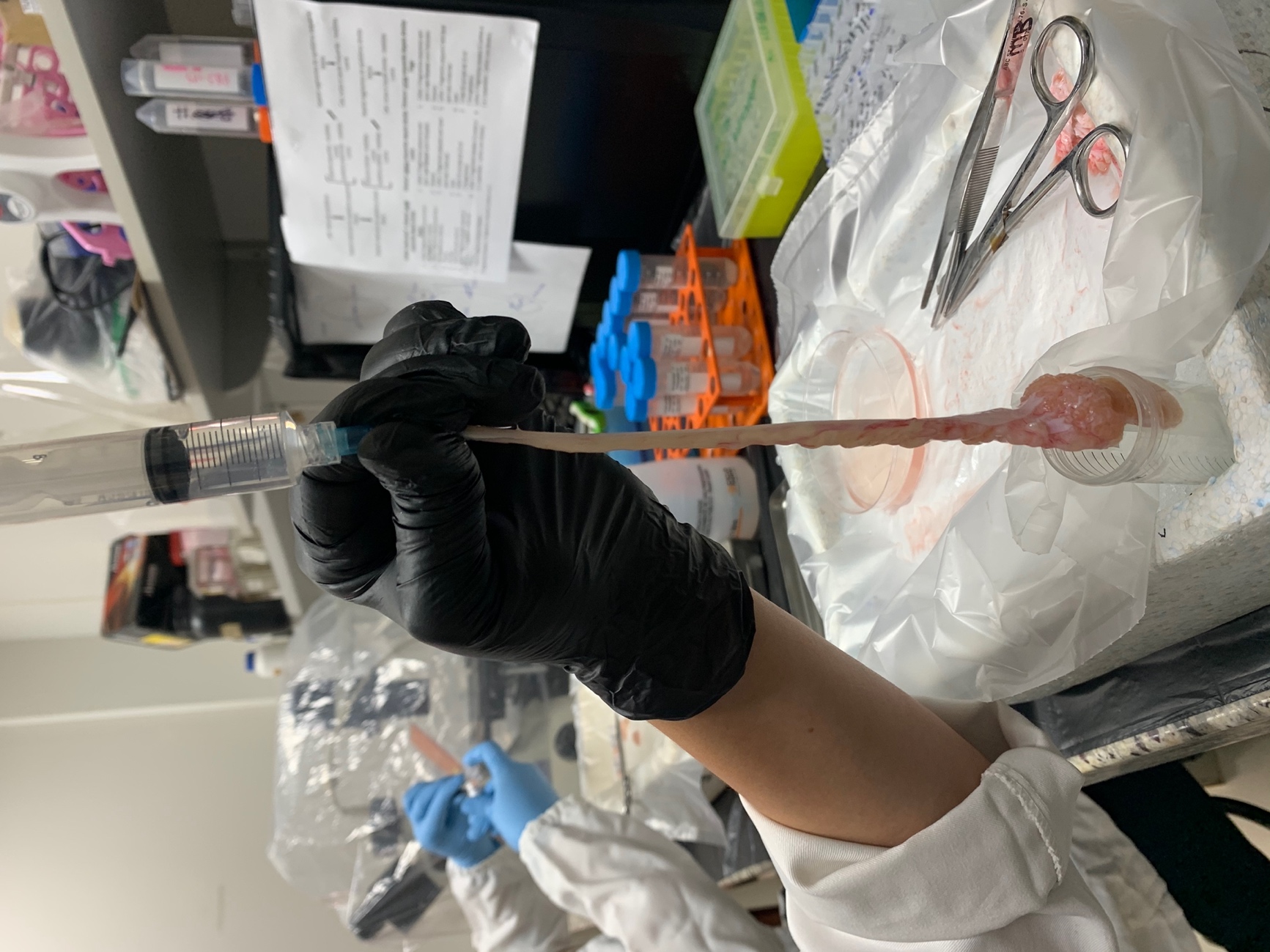
**

**Supplementary Figure S3. Representative gates of sperm-epididymossomes (epEVs) interaction evaluated on flow cytometry.** In the first gate, it is represented the population of events in which is expected to be sperm (called P1). In the second gate, it is represented the positive events for Hoechst 33342 (called SPERM). In the third gate, the minor bar represents the percentage of population Sperm that is positive to green fluorescence (called Sperm + EVs). The major bar represents all the population of Sperm (called Sperm with green fluorescence) and was used to calculate the median of green fluorescence per event. In the representative images, the gates of the top represent the Control group treated with PBS stained with green-marker, and the gates of the bottom represent the epEVs group treated with 1,000 epEVs/sperm. The period of incubation was 3 hours. The epEVs were stained with a green marker (PKH67^®^) before incubation with sperm.

**
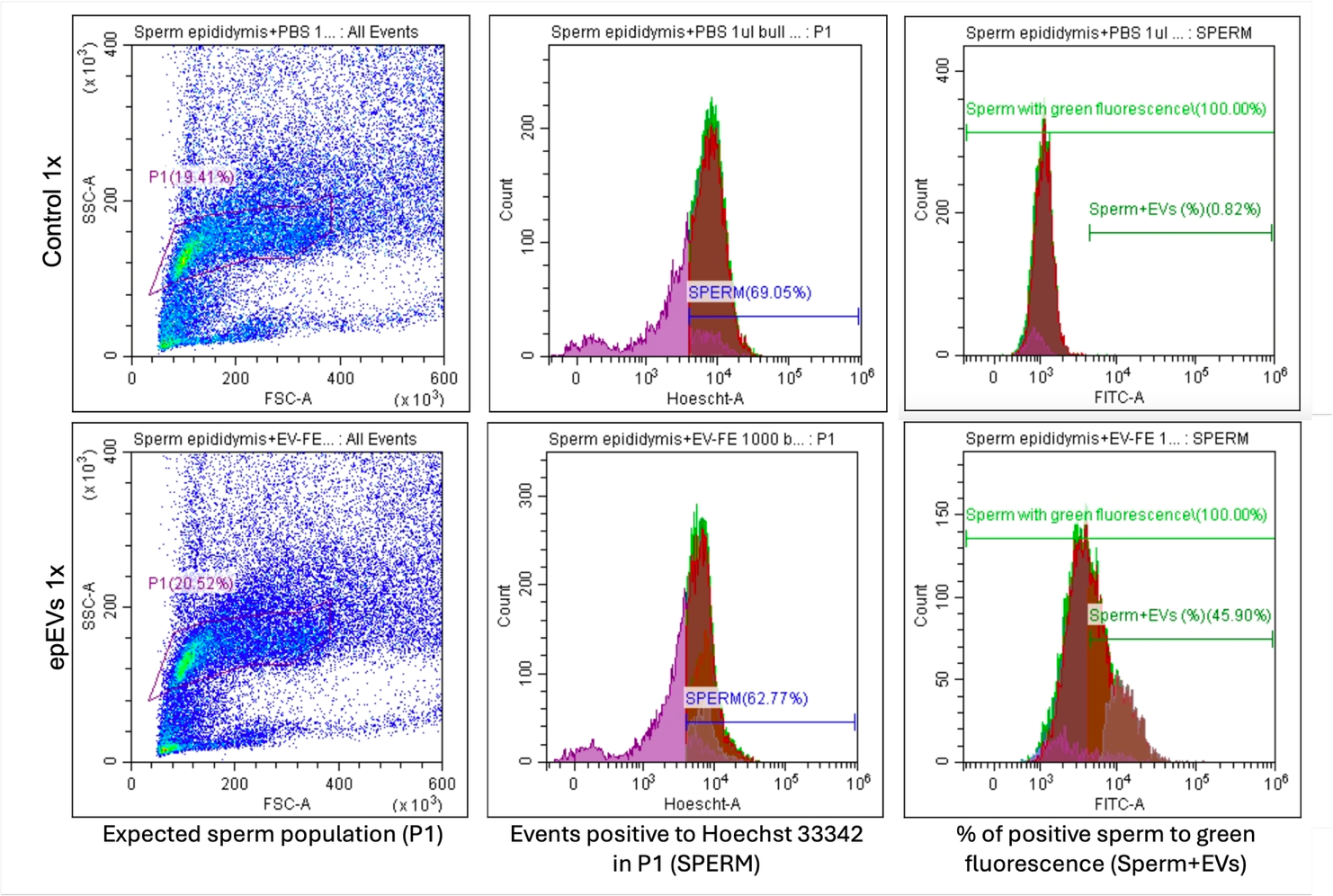
**

**Supplementary Video S1. Retrograde perfusion of epididymal cauda.** A 22G catheter connected to a 10 mL syringe was introduced into the deferent duct and PBS was injected to perfuse the epididymal *cauda* duct. In the video it is possible to visualize the entrance of PBS in the epididymal tubule and the obtention of epididymal fluid.

**VIDEO FILE S1**

**Supplementary Video S2. Time-lapse assessment of interaction between sperm and epididymosomes (epEVs).** A ratio of 1,000 epEVs/sperm was incubated with post-thawed epididymal sperm in Talp-Sperm media at the same conditions of the experimental sets. For the assessment of the interaction, epEVs were stained with PKH67^®^ Green Fluorescent Cell Linker and incubated with selected epididymal sperm that was stained with 1 µl of Hoechst 33342 0.05 mg/mL. After, 5 μl of the sample was added to a glass and covered with a coverslip. Time-lapse was performed using a Thunder Imager 3D Assay^®^ (Leica, Germany) fluorescence microscopy with objective of 63x and the Leica LAS X Software that was configured to acquire images at 4-second intervals, generating consecutive 15-minute videos over a total observation period of 3 hours to monitor sperm-epEVs interaction.

**VIDEO FILE S2**

**Supplementary Video S3. Excerpt from the time-lapse assessment (Video S2) highlighting the interaction between sperm and epididymosomes (epEVs).** A cropped region of the video shows a motile sperm interacting with epEVs. The dashed white arrow indicates when this sperm cell initiates the motility movement in the assessed field, and the solid white arrow indicates the moment that this sperm cell stops the motility movement. For the assessment, epEVs were stained with PKH67^®^ Green Fluorescent Cell Linker and incubated with selected epididymal sperm that was stained with 1 µl of Hoechst 33342 0.05 mg/mL. Time-lapse was performed using a Thunder Imager 3D Assay^®^ (Leica, Germany) fluorescence microscopy with objective of 63x and the Leica LAS X Software that was configured to acquire images at 4-second intervals, generating consecutive 15-minute videos.

**VIDEO FILE S3**

**Supplementary Video S4. Excerpt from the time-lapse assessment (Video S2) highlighting the interaction between sperm and epididymosomes (epEVs).** The dashed white arrows were included in the video to indicate when epEVs passed through sperm without effectively interacting with them. The solid white arrows were included to indicate when epEVs are interacting with sperm. For the assessment, epEVs were stained with PKH67^®^ Green Fluorescent Cell Linker and incubated with selected epididymal sperm that was stained with 1 µl of Hoechst 33342 0.05 mg/mL. Time-lapse was performed using a Thunder Imager 3D Assay^®^ (Leica, Germany) fluorescence microscopy with objective of 63x and the Leica LAS X Software that was configured to acquire images at 4-second intervals, generating consecutive 15-minute videos.

**VIDEO FILE S4**

**Supplementary Table S1. Presence of TOM20, a marker of mitochondria, in sperm.** Data are shown as events/μL and median of fluorescence per event of the events positive for TOM20. Sperm were used as positive control for this antibody.

| **Sample** | **Events/μL** | **Fluorescence/event** |
| --- | --- | --- |
| Control sperm^1^ | 1,286.5 | 0.13 |
| TOM20 sperm^2^ | 9,816.6 | 1.05 |

^1,2^Control sperm and TOM20 sperm are respectively sperm sample that was not incubated with TOM20 and sperm sample that was incubated with TOM20.

**Supplementary Table S2. List of the 380 microRNAs investigated in the epEVs.** List of the microRNAs analyzed in RNA extracted from epEVs.

|  | **miRNA** | **Primer Sequence (5' - 3')** |
| --- | --- | --- |
|  | bta-let-7a-3p | CTATACAATCTACTGTCTTTC |
|  | bta-miR-103 | AGCAGCATTGTACAGGGCTATGA |
|  | bta-let-7a-5p | TGAGGTAGTAGGTTGTATAGTT |
|  | bta-miR-105a | TCAAATGCTCAGACTCCTGTGGT |
|  | bta-let-7b | TGAGGTAGTAGGTTGTGTGGTT |
|  | bta-miR-105b | TCAAATGCTCAGACTCCTTGGT |
|  | bta-let-7c | TGAGGTAGTAGGTTGTATGGTT |
|  | bta-miR-106a | AAAAGTGCTTACAGTGCAGGTA |
|  | bta-let-7d | AGAGGTAGTAGGTTGCATAGTT |
|  | bta-miR-106b | TAAAGTGCTGACAGTGCAGAT |
|  | bta-let-7e | TGAGGTAGGAGGTTGTATAGT |
|  | bta-miR-107 | AGCAGCATTGTACAGGGCTATC |
|  | bta-let-7f | TGAGGTAGTAGATTGTATAGTT |
|  | bta-miR-10a | TACCCTGTAGATCCGAATTTGTG |
|  | bta-let-7g | TGAGGTAGTAGTTTGTACAGTT |
|  | bta-miR-10b | TACCCTGTAGAACCGAATTTGTG |
|  | bta-let-7i | TGAGGTAGTAGTTTGTGCTGTT |
|  | bta-miR-122 | TGGAGTGTGACAATGGTGTTTG |
|  | bta-miR-1 | TGGAATGTAAAGAAGTATGTAT |
|  | bta-miR-124a | TAAGGCACGCGGTGAATGCCAAG |
|  | bta-miR-100 | AACCCGTAGATCCGAACTTGTG |
|  | bta-miR-124b | TAAGGCACGCGGTGAATGCCAAG |
|  | bta-miR-101 | TACAGTACTGTGATAACTGAA |
|  | bta-miR-125a | TCCCTGAGACCCTTTAACCTGTG |
|  | bta-miR-125b | TCCCTGAGACCCTAACTTGTGA |
|  | bta-miR-133b | TTTGGTCCCCTTCAACCAGCTA |
|  | bta-miR-126-3p | CGTACCGTGAGTAATAATGCG |
|  | bta-miR-133c | ATTTGGTTCCATTTTACCAGC |
|  | bta-miR-126-5p | CATTATTACTTTTGGTACGCG |
|  | bta-miR-134 | TGTGACTGGTTGACCAGAGTGG |
|  | bta-miR-127 | TCGGATCCGTCTGAGCTTGGCT |
|  | bta-miR-135a | TATGGCTTTTTATTCCTATGTGA |
|  | bta-miR-128 | TCACAGTGAACCGGTCTCTTT |
|  | bta-miR-135b | TATGGCTTTTCATTCCTATGTGA |
|  | bta-miR-129 | CTTTTTGCGGTCTGGGCTTGCT |
|  | bta-miR-136 | ACTCCATTTGTTTTGATGATGGA |
|  | bta-miR-129-3p | AAGCCCTTACCCCAAAAAGCAT |
|  | bta-miR-137 | TTATTGCTTAAGAATACGCGTAG |
|  | bta-miR-129-5p | CTTTTTGCGGTCTGGGCTTGCT |
|  | bta-miR-138 | AGCTGGTGTTGTGAATCAGGCCG |
|  | bta-miR-130a | CAGTGCAATGTTAAAAGGGCAT |
|  | bta-miR-139 | TCTACAGTGCACGTGTCTCCAGT |
|  | bta-miR-130b | CAGTGCAATGATGAAAGGGCAT |
|  | bta-miR-140 | TACCACAGGGTAGAACCACGGA |
|  | bta-miR-132 | TAACAGTCTACAGCCATGGTCG |
|  | bta-miR-141 | TAACACTGTCTGGTAAAGATGG |
|  | bta-miR-133a | TTTGGTCCCCTTCAACCAGCTG |
|  | bta-miR-142-3p | AGTGTTTCCTACTTTATGGATG |
|  | bta-miR-142-5p | CATAAAGTAGAAAGCACTAC |
|  | bta-miR-151-3p | CTAGACTGAAGCTCCTTGAGG |
|  | bta-miR-143 | TGAGATGAAGCACTGTAGCTCG |
|  | bta-miR-151-5p | TCGAGGAGCTCACAGTCTAGT |
|  | bta-miR-144 | TACAGTATAGATGATGTACTAG |
|  | bta-miR-152 | TCAGTGCATGACAGAACTTGGG |
|  | bta-miR-145 | GTCCAGTTTTCCCAGGAATCCCT |
|  | bta-miR-153 | TTGCATAGTCACAAAAGTGATC |
|  | bta-miR-146a | TGAGAACTGAATTCCATAGGTTGT |
|  | bta-miR-154a | TAGGTTATCCGTGTAGCCTTCG |
|  | bta-miR-146b | TGAGAACTGAATTCCATAGGCTGT |
|  | bta-miR-154b | AGAGGTCTTCCATGGTGCATTCG |
|  | bta-miR-147 | GTGTGCGGAAATGCTTCTGCTA |
|  | bta-miR-154c | AGATATTGCACGGTTGATCTCT |
|  | bta-miR-148a | TCAGTGCACTACAGAACTTTGT |
|  | bta-miR-155 | TTAATGCTAATCGTGATAGGGGT |
|  | bta-miR-148b | TCAGTGCATCACAGAACTTTGT |
|  | bta-miR-15a | TAGCAGCACATAATGGTTTGT |
|  | bta-miR-149-3p | GAGGGAGGGACGGGGGCTGTGC |
|  | bta-miR-15b | TAGCAGCACATCATGGTTTACA |
|  | bta-miR-149-5p | TCTGGCTCCGTGTCTTCACTCCC |
|  | bta-miR-16a | TAGCAGCACGTAAATATTGGTG |
|  | bta-miR-150 | TCTCCCAACCCTTGTACCAGTGT |
|  | bta-miR-16b | TAGCAGCACGTAAATATTGGC |
|  | bta-miR-17-3p | ACTGCAGTGAAGGCACTTGT |
|  | bta-miR-188 | CATCCCTTGCATGGTGGAGGGT |
|  | bta-miR-17-5p | CAAAGTGCTTACAGTGCAGGTAGT |
|  | bta-miR-18a | TAAGGTGCATCTAGTGCAGATA |
|  | bta-miR-181a | AACATTCAACGCTGTCGGTGAGTT |
|  | bta-miR-18b | TAAGGTGCATCTAGTGCAGTTA |
|  | bta-miR-181b | AACATTCATTGCTGTCGGTGGGTT |
|  | bta-miR-190a | TGATATGTTTGATATATTAGGT |
|  | bta-miR-181c | AACATTCAACCTGTCGGTGAGTTT |
|  | bta-miR-190b | TGATATGTTTGATATTGGGTT |
|  | bta-miR-181d | AACATTCATTGTTGTCGGTGGGT |
|  | bta-miR-191 | CAACGGAATCCCAAAAGCAGCTG |
|  | bta-miR-182 | TTTGGCAATGGTAGAACTCACACT |
|  | bta-miR-192 | CTGACCTATGAATTGACAGCCAG |
|  | bta-miR-183 | TATGGCACTGGTAGAATTCACTG |
|  | bta-miR-193a | GGGACTTTGTAGGCCAGTT |
|  | bta-miR-184 | TGGACGGAGAACTGATAAGGGT |
|  | bta-miR-193a-3p | AACTGGCCTACAAAGTCCCAGT |
|  | bta-miR-185 | TGGAGAGAAAGGCAGTTCCTGA |
|  | bta-miR-193a-5p | TGGGTCTTTGCGGGCGAGATGA |
|  | bta-miR-186 | CAAAGAATTCTCCTTTTGGGCT |
|  | bta-miR-193b | AACTGGCCCACAAAGTCCCGCTTT |
|  | bta-miR-187 | TCGTGTCTTGTGTTGCAGCCGG |
|  | bta-miR-194 | TGTAACAGCAACTCCATGTGGA |
|  | bta-miR-195 | TAGCAGCACAGAAATATTGGCA |
|  | bta-miR-200c | TAATACTGCCGGGTAATGATGGA |
|  | bta-miR-196a | TAGGTAGTTTCATGTTGTTGGG |
|  | bta-miR-202 | TTCCTATGCATATACTTCTTT |
|  | bta-miR-196b | TAGGTAGTTTCCTGTTGTTGGGA |
|  | bta-miR-204 | TTCCCTTTGTCATCCTATGCCT |
|  | bta-miR-197 | TTCACCACCTTCTCCACCCAGC |
|  | bta-miR-205 | TCCTTCATTCCACCGGAGTCTG |
|  | bta-miR-199a-3p | ACAGTAGTCTGCACATTGGTTA |
|  | bta-miR-206 | TGGAATGTAAGGAAGTGTGTGG |
|  | bta-miR-199a-5p | CCCAGTGTTCAGACTACCTGTT |
|  | bta-miR-208a | ATAAGACGAGCAAAAAGCTTGT |
|  | bta-miR-199b | CCCAGTGTTTAGACTATCTGTTC |
|  | bta-miR-208b | ATAAGACGAACAAAAGGTTTGT |
|  | bta-miR-199c | TACAGTAGTCTGCACATTGG |
|  | bta-miR-20a | TAAAGTGCTTATAGTGCAGGTAG |
|  | bta-miR-19a | TGTGCAAATCTATGCAAAACTGA |
|  | bta-miR-20b | CAAAGTGCTCACAGTGCAGGTA |
|  | bta-miR-19b | TGTGCAAATCCATGCAAAACTGA |
|  | bta-miR-21-3p | AACAGCAGTCGATGGGCTGTCT |
|  | bta-miR-200a | TAACACTGTCTGGTAACGATGTT |
|  | bta-miR-21-5p | TAGCTTATCAGACTGATGTTGACT |
|  | bta-miR-200b | TAATACTGCCTGGTAATGATG |
|  | bta-miR-210 | ACTGTGCGTGTGACAGCGGCTGA |
|  | bta-miR-211 | TTCCCTTTGTCATCCTTTGCC |
|  | bta-miR-22-5p | AGTTCTTCAGTGGCAAGCTTTA |
|  | bta-miR-212 | ACCTTGGCTCTAGACTGCTTACT |
|  | bta-miR-221 | AGCTACATTGTCTGCTGGGTTT |
|  | bta-miR-214 | ACAGCAGGCACAGACAGGCAGT |
|  | bta-miR-222 | AGCTACATCTGGCTACTGGGT |
|  | bta-miR-215 | ATGACCTATGAATTGACAGACA |
|  | bta-miR-223 | TGTCAGTTTGTCAAATACCCCA |
|  | bta-miR-216a | TAATCTCAGCTGGCAACTGTGA |
|  | bta-miR-224 | CAAGTCACTAGTGGTTCCGTTTA |
|  | bta-miR-216b | AAATCTCTGCAGGCAAATGTGA |
|  | bta-miR-23a | ATCACATTGCCAGGGATTTCCA |
|  | bta-miR-217 | TACTGCATCAGGAACTGATTGGAT |
|  | bta-miR-23b-3p | ATCACATTGCCAGGGATTACCAC |
|  | bta-miR-218 | TTGTGCTTGATCTAACCATGTG |
|  | bta-miR-23b-5p | GGGTTCCTGGCATGCTGATTT |
|  | bta-miR-219 | AGAGTTGAGTCTGGACGTCCCG |
|  | bta-miR-24 | GTGCCTACTGAGCTGATATCAGT |
|  | bta-miR-219-3p | AGAATTGTGGCTGGACATCTG |
|  | bta-miR-24-3p | TGGCTCAGTTCAGCAGGAACAG |
|  | bta-miR-219-5p | TGATTGTCCAAACGCAATTCTT |
|  | bta-miR-25 | CATTGCACTTGTCTCGGTCTGA |
|  | bta-miR-22-3p | AAGCTGCCAGTTGAAGAACTG |
|  | bta-miR-26a | TTCAAGTAATCCAGGATAGGCT |
|  | bta-miR-26b | TTCAAGTAATTCAGGATAGGTT |
|  | bta-miR-29d-3p | TAGCACCATTTGAAATCGATTA |
|  | bta-miR-26c | AGCCTATCCTGGATTACTTGAA |
|  | bta-miR-29d-5p | TGACCGATTTCTCCTGGTGTT |
|  | bta-miR-27a-3p | TTCACAGTGGCTAAGTTCCG |
|  | bta-miR-29e | TAGCATCATTTGAAATCAGTGTTT |
|  | bta-miR-27a-5p | AGGGCTTAGCTGCTTGTGAGCA |
|  | bta-miR-301a | CAGTGCAATAGTATTGTCAAAGCAT |
|  | bta-miR-27b | TTCACAGTGGCTAAGTTCTGC |
|  | bta-miR-301b | CAGTGCAATGATATTGTCAAAGCAT |
|  | bta-miR-28 | AAGGAGCTCACAGTCTATTGAG |
|  | bta-miR-302a | AAGTGCTTCCATGTTTTAGTGA |
|  | bta-miR-296-3p | GAGGGTTGGGCGGAGGCTTTCC |
|  | bta-miR-302b | TAAGTGCTTCCATGTTTTAGTAG |
|  | bta-miR-296-5p | GAGGGCCCCCCCCAATCCT |
|  | bta-miR-302c | TAAGTGCTTCCATGTTTCAGTGG |
|  | bta-miR-299 | TGGTTTACCGTCCCACATACAT |
|  | bta-miR-302d | TAAGTGCTTCCATGTTTTAGT |
|  | bta-miR-29a | CTAGCACCATCTGAAATCGGTTA |
|  | bta-miR-3064 | TTGCCACACTGCAACACCTTACA |
|  | bta-miR-29b | TAGCACCATTTGAAATCAGTGTT |
|  | bta-miR-30a-5p | TGTAAACATCCTCGACTGGAAGCT |
|  | bta-miR-29c | TAGCACCATTTGAAATCGGTTA |
|  | bta-miR-30b-3p | CTGGGAGGTGGATGTTTACTT |
|  | bta-miR-30b-5p | TGTAAACATCCTACACTCAGCT |
|  | bta-miR-328 | CTGGCCCTCTCTGCCCTTCCGT |
|  | bta-miR-30c | TGTAAACATCCTACACTCTCAGC |
|  | bta-miR-329a | AACACACCTGGTTAACCTTTTT |
|  | bta-miR-30d | TGTAAACATCCCCGACTGGAAGCT |
|  | bta-miR-329b | AGAGGTTTTCTGGGTTTCTGTTT |
|  | bta-miR-30e-5p | TGTAAACATCCTTGACTGGAAGCT |
|  | bta-miR-330 | GCAAAGCACACGGCCTGCAGAGA |
|  | bta-miR-30f | TGTAAACACCCTACACTCTCAGCT |
|  | bta-miR-331-3p | GCCCCTGGGCCTATCCTAGAA |
|  | bta-miR-31 | AGGCAAGATGCTGGCATAGCT |
|  | bta-miR-331-5p | TCTAGGTATGGTCCCAGG |
|  | bta-miR-32 | TATTGCACATGACTAAGTTGCAT |
|  | bta-miR-335 | TCAAGAGCAATAACGAAAAATGT |
|  | bta-miR-320a | AAAAGCTGGGTTGAGAGGGCGA |
|  | bta-miR-338 | TCCAGCATCAGTGATTTTGTTGA |
|  | bta-miR-320b | AGCTGGGTTGAGAGGGTGGT |
|  | bta-miR-339a | TCCCTGTCCTCCAGGAGCTCAC |
|  | bta-miR-323 | GCACATTACACGGTCGACCTCT |
|  | bta-miR-339b | TCCCTGTCCTCCAGGAGCTC |
|  | bta-miR-324 | CGCATCCCCTAGGGCATTGGTGT |
|  | bta-miR-33a | GTGCATTGTAGTTGCATTGCA |
|  | bta-miR-326 | CCTCTGGGCCCTTCCTCCAG |
|  | bta-miR-33b | GTGCATTGCTGTTGCATTGC |
|  | bta-miR-340 | TCCGTCTCAGTTACTTTATAGCC |
|  | bta-miR-365-3p | TAATGCCCCTAAAAATCCTTAT |
|  | bta-miR-342 | TCTCACACAGAAATCGCACCCATCT |
|  | bta-miR-365-5p | AGGGACTTTTGGGGGCAGATGTG |
|  | bta-miR-345-3p | CCTGAACTAGGGGTCTGGAG |
|  | bta-miR-367 | GAATTGCACTTTAGCAATGGTGA |
|  | bta-miR-345-5p | GCTGACTCCTAGTCCAGTGCT |
|  | bta-miR-369-3p | AATAATACATGGTTGATCTTT |
|  | bta-miR-346 | TGTCTGCCCGCATGCCTGCCTCT |
|  | bta-miR-369-5p | ATCGACCGTGTTATATTCGC |
|  | bta-miR-34a | TGGCAGTGTCTTAGCTGGTTGT |
|  | bta-miR-370 | GCCTGCTGGGGTGGAACCTGGT |
|  | bta-miR-34b | AGGCAGTGTAATTAGCTGATTG |
|  | bta-miR-371 | AAGTGCCGCCATGTTTTGAGTGT |
|  | bta-miR-34c | AGGCAGTGTAGTTAGCTGATTG |
|  | bta-miR-374a | TTATAATACAACCTGATAAGTG |
|  | bta-miR-361 | TTATCAGAATCTCCAGGGGTAC |
|  | bta-miR-374b | ATATAATACAACCTGCTAAGTG |
|  | bta-miR-362-3p | AACACACCTATTCAAGGATTC |
|  | bta-miR-375 | TTTTGTTCGTTCGGCTCGCGTGA |
|  | bta-miR-362-5p | AATCCTTGGAACCTAGGTGTGAGT |
|  | bta-miR-376a | ATCATAGAGGAAAATCCACGT |
|  | bta-miR-363 | ATTGCACGGTATCCATCTGCG |
|  | bta-miR-376b | ATCATAGAGGAAAATCCATGTT |
|  | bta-miR-376c | GTGGATATTCCTTCTATGTTTA |
|  | bta-miR-382 | GAAGTTGTTCGTGGTGGATTCG |
|  | bta-miR-376d | ATCATAGAGGAAAATCCACAT |
|  | bta-miR-383 | AGATCAGAAGGTGATTGTGGCT |
|  | bta-miR-376e | AACATAGAGGAAAATCCACATT |
|  | bta-miR-409a | AGGTTACCCGAGCAACTTTGCAT |
|  | bta-miR-377 | ATCACACAAAGGCAACTTTTGT |
|  | bta-miR-409b | GGGGTTCACCGAGCAACATTC |
|  | bta-miR-378 | ACTGGACTTGGAGTCAGAAGGC |
|  | bta-miR-410 | AATATAACACAGATGGCCTGT |
|  | bta-miR-378b | ACTTGACTTGGAGTCAGAAGGC |
|  | bta-miR-411a | ATAGTAGACCGTATAGCGTACG |
|  | bta-miR-378c | ACTGGACTTGGAGTCAGAAGT |
|  | bta-miR-411b | TGGTCGACCATAAAACGTACGT |
|  | bta-miR-378d | CTGGACTTGGAGTCAGAAGACC |
|  | bta-miR-411c-3p | TGTATGTCAACTGATCCACAGT |
|  | bta-miR-379 | TGGTAGACTATGGAACGTAGG |
|  | bta-miR-411c-5p | GGTTGATCAGAGAACATACATT |
|  | bta-miR-380-3p | TATGTAATGTGGTCCACGTCT |
|  | bta-miR-412 | ACTTCACCTGGTCCACTAGCTGT |
|  | bta-miR-380-5p | TGGTTGACCATAGAACATGCGC |
|  | bta-miR-421 | ATCAACAGACATTAATTGGGCGC |
|  | bta-miR-381 | TATACAAGGGCAAGCTCTCTGT |
|  | bta-miR-423-3p | AAGCTCGGTCTGAGGCCCCTCAGT |
|  | bta-miR-423-5p | TGAGGGGCAGAGAGCGAGACTTT |
|  | bta-miR-449c | AGGCAGTGCATCTCTAGCTGG |
|  | bta-miR-424-3p | CAAAACGTGAGGCGCTGCTAT |
|  | bta-miR-449d | GAAGGCTGTGTGCTGTGGAG |
|  | bta-miR-424-5p | CAGCAGCAATTCATGTTTTGA |
|  | bta-miR-450a | TTTTGCGATGTGTTCCTAATAT |
|  | bta-miR-425-3p | ATCGGGAATGTCGTGTCCGCCC |
|  | bta-miR-450b | TTTTGCAATATGTTCCTGAATA |
|  | bta-miR-425-5p | ATGACACGATCACTCCCGTTGA |
|  | bta-miR-451 | AAACCGTTACCATTACTGAGTTT |
|  | bta-miR-429 | TAATACTGTCTGGTAATGCCGT |
|  | bta-miR-452 | TGTTTGCAGAGGAAACTGAGAC |
|  | bta-miR-431 | TGTCTTGCAGGCCGTCATGCAGG |
|  | bta-miR-4523 | GACCGAGAGGGCCTCGGCTGT |
|  | bta-miR-432 | TCTTGGAGTAGGTCATTGGGTGG |
|  | bta-miR-453 | AGGTTGTCCGTGGTGAGTTCGCA |
|  | bta-miR-433 | ATCATGATGGGCTCCTCGGTGT |
|  | bta-miR-454 | TAGTGCAATATTGCTTATAGGGT |
|  | bta-miR-448 | TTGCATATGTAGGATGTCCCAT |
|  | bta-miR-455-3p | GCAGTCCATGGGCATATACACT |
|  | bta-miR-449a | TGGCAGTGTATTGTTAGCTGGT |
|  | bta-miR-455-5p | TATGTGCCTTTGGACTACATC |
|  | bta-miR-449b | AGGCAGTGTATTGTTAGCTGGC |
|  | bta-miR-483 | TCACTCCTCTCCTCCCGTCTT |
|  | bta-miR-484 | TCAGGCTCAGTCCCCTCCCGAT |
|  | bta-miR-496 | TGAGTATTACATGGCCAATCTC |
|  | bta-miR-485 | AGAGGCTGGCCGTGATGAATTCG |
|  | bta-miR-497 | CAGCAGCACACTGTGGTTTGTA |
|  | bta-miR-486 | TCCTGTACTGAGCTGCCCCGAG |
|  | bta-miR-499 | TTAAGACTTGCAGTGATGTTT |
|  | bta-miR-487a | AATCATACAGGGACATCCAGT |
|  | bta-miR-500 | TAATCCTTGCTACCTGGGTGAGA |
|  | bta-miR-487b | AATCGTACAGGGTCATCCACTT |
|  | bta-miR-502a | AATGCACCTGGGCAAGGATTCA |
|  | bta-miR-488 | TTGAAAGGCTGTTTCTTGGTC |
|  | bta-miR-502b | AATCCACCTGGGCAAGGATTC |
|  | bta-miR-489 | GTGACATCACATATATGGCGAC |
|  | bta-miR-503-3p | GGAGTATTGTTTCTGCTGCCCGG |
|  | bta-miR-490 | CAACCTGGAGGACTCCATGCTG |
|  | bta-miR-503-5p | TAGCAGCGGGAACAGTACTG |
|  | bta-miR-491 | AGTGGGGAACCCTTCCATGAGG |
|  | bta-miR-504 | AGACCCTGGTCTGCACTCTGTC |
|  | bta-miR-493 | TGAAGGTCTACTGTGTGCCAGG |
|  | bta-miR-505 | CGTCAACACTTGCTGGTTTCCT |
|  | bta-miR-494 | TGAAACATACACGGGAAACCTC |
|  | bta-miR-532 | CATGCCTTGAGTGTAGGACCGT |
|  | bta-miR-495 | AAACAAACATGGTGCACTTCTT |
|  | bta-miR-539 | GGAGAAATTATCCTTGGTGTGT |
|  | bta-miR-541 | TGGTGGGCACAGAATCCGGCCT |
|  | bta-miR-582 | TTACAGTTGTTCAACCAGTTACT |
|  | bta-miR-542-5p | TCGGGGATCATCATGTCACGAG |
|  | bta-miR-584 | TGGTTTGCCTGGGACTGAG |
|  | bta-miR-543 | AAACATTCGCGGTGCACTTCTT |
|  | bta-miR-592 | ATTGTGTCAATATGCGATGATGT |
|  | bta-miR-544a | ATTCTGCATTTTTAGCAAGTTC |
|  | bta-miR-599 | GTTGTGTCAGTTTATCAAAC |
|  | bta-miR-544b | ATTCTGCATTTCTAACAAGTTC |
|  | bta-miR-615 | GGGGGTCCCCGGTGCTCGGATC |
|  | bta-miR-545-3p | ATCAACAAACATTTATTGTGTG |
|  | bta-miR-628 | ATGCTGACATATTTACTAGAGG |
|  | bta-miR-545-5p | TCAGTAAATGTTTATTGGATG |
|  | bta-miR-631 | AGACCTGGCTTAGACCTCAGC |
|  | bta-miR-551a | GCGACCCAATCTTGGTTTCCA |
|  | bta-miR-652 | AATGGCGCCACTAGGGTTGTG |
|  | bta-miR-551b | GGCGACCCATACTTGGTTTCAG |
|  | bta-miR-653 | GTGTTGAAACAATCTCTGTTG |
|  | bta-miR-562 | AAAGCAGCTGTACCATTTAC |
|  | bta-miR-654 | TATGTCTGCTGACCATCACCTT |
|  | bta-miR-568 | ATGTATAAATGTATACACAC |
|  | bta-miR-655 | ATAATACATGGTTAACCTCTCT |
|  | bta-miR-574 | TGAGTGTGTGTGTGTGAGTGTGTG |
|  | bta-miR-656 | AATATTATACAGTCAACCTCT |
|  | bta-miR-658 | GGCGGAGGGAAGCGGGTCCGTTGGT |
|  | bta-miR-758 | TTTGTGACCTGGTCCACTAACC |
|  | bta-miR-660 | TACCCATTGCATATCGGAGCTG |
|  | bta-miR-759 | GCAGACTGCAAACAATTTTGAC |
|  | bta-miR-664a | CAGGCTGGGGTGTGTGTGGATG |
|  | bta-miR-760-3p | CGGCTCTGGGTCTGTGGGGA |
|  | bta-miR-664b | TATTCATTTATCTCCCAGCCTAC |
|  | bta-miR-760-5p | CCCCTCAGTCCACCAGAGCCCG |
|  | bta-miR-665 | ACCAGTAGGCCGAGGCCCCT |
|  | bta-miR-761 | GCAGCAGGGTGAAACTGACACA |
|  | bta-miR-669 | TGTGGGTGTGTGCATGTGCGTG |
|  | bta-miR-763 | CCAGCTGGGAGGAACCAGTGGC |
|  | bta-miR-670 | TCCCTGAGTATATGTGGTGAA |
|  | bta-miR-764 | GGTGCTCACTCGTCCTTCT |
|  | bta-miR-671 | AGGAAGCCCTGGAGGGGCTGGAG |
|  | bta-miR-767 | TGCACCATGGTTGTCTGAGCATG |
|  | bta-miR-677 | CTCACTGATGAGCAGCTTCTGAC |
|  | bta-miR-769 | TGAGACCTCCGGGTTCTGAGCT |
|  | bta-miR-7 | TGGAAGACTAGTGATTTTGTTGTT |
|  | bta-miR-873 | GCAGGAACTTGTGAGTCTCCT |
|  | bta-miR-708 | AAGGAGCTTACAATCTAGCTGGG |
|  | bta-miR-874 | CTGCCCTGGCCCGAGGGACCGA |
|  | bta-miR-744 | TGCGGGGCTAGGGCTAACAGCA |
|  | bta-miR-875 | TATACCTCAGTTTTATCAGGTG |
|  | bta-miR-876 | TGGATTTCTTTGTGAATCACCA |
|  | bta-miR-98 | TGAGGTAGTAAGTTGTATTGTT |
|  | bta-miR-877 | GTAGAGGAGATGGCGCAGGG |
|  | bta-miR-99a-3p | CAAGCTCGCTTCTATGGGT |
|  | bta-miR-885 | TCCATTACACTACCCTGCCTCT |
|  | bta-miR-99a-5p | AACCCGTAGATCCGATCTTGT |
|  | bta-miR-9-3p | ATAAAGCTAGATAACCG |
|  | bta-miR-99b | CACCCGTAGAACCGACCTTGCG |
|  | bta-miR-9-5p | TCTTTGGTTATCTAGCTGTATG |
|  | bta-miR-1179 | AAGCATTCTTTCATTGGTTGG |
|  | bta-miR-92a | TATTGCACTTGTCCCGGCCTGT |
|  | bta-miR-1185 | AGAGGATACCCTTTGTATGTT |
|  | bta-miR-92b | TATTGCACTCGTCCCGGCCTCC |
|  | bta-miR-1193 | TAGGTCACCCGTTTGACTATC |
|  | bta-miR-93 | CAAAGTGCTGTTCGTGCAGGTA |
|  | bta-miR-1197 | TAGGACACATGGTCTACTTCT |
|  | bta-miR-935 | CCAGTTACCGCTTCCGCTACCGC |
|  | bta-miR-122 | TGGAGTGTGACAATGGTGTTTG |
|  | bta-miR-940 | AAGGCTGGGCCCCCGCTCCGC |
|  | bta-miR-1224 | GTGAGGACTCGGGAGGTGGAG |
|  | bta-miR-95 | TTCAACGGGTATTTATTGAGCA |
|  | bta-miR-1225-3p | CCGAGCCCCTGTGCCGCCCCCAG |
|  | bta-miR-96 | TTTGGCACTAGCACATTTTTGCT |
|  | bta-miR-1246 | AATGGATTTTTGGAGCAGG |
|  | bta-miR-1247-3p | CGGGAACGTCGGGACTGGAGC |
|  | bta-miR-1296 | TTAGGGCCCTGGCTCCATCTCC |
|  | bta-miR-1247-5p | ACCCGTCCCGTGCGTCCCCGGA |
|  | bta-miR-1298 | TTCATTCGGCTGTCCAGATGTA |
|  | bta-miR-1248 | ACCTTCTTGTATAAGCACTGTGCTAAA |
|  | bta-miR-1301 | TTGCAGCTGCCTAGGAGTGATTTC |
|  | bta-miR-1249 | ACGCCCTTCCCCCCCTTCTTCA |
|  | bta-miR-1306 | CCACCTCCCCTGCAAACGTCC |
|  | bta-miR-1260b | ATCCCACCACTGCCACCA |
|  | bta-miR-1307 | ACTCGGCGTGGCGTCGGTCGTG |
|  | bta-miR-1271 | CTTGGCACCTAGTAAGTACTCA |
|  | bta-miR-1343-3p | CTCCTGGGGCCCGCACTCTC |
|  | bta-miR-1277 | TACGTAGATATATATGTATTTT |
|  | bta-miR-1343-5p | TGGGGAGCGGCCCCCGGGCGGG |
|  | bta-miR-1281 | TCGCCTCCTCCTCTCCC |
|  | bta-miR-1388-3p | ATCTCAGGTTTGTCAGCCCGCA |
|  | bta-miR-1282 | TCGTTTGCCTTTTTCTGCTT |
|  | bta-miR-1284 | TCTGCACAGACCCTGGCTTTTC |
|  | bta-miR-1287 | TGCTGGATCAGTGGTTTGAGTC |
|  | bta-miR-1291 | TGGCCCTGACTGAAGACCTGCAGT |
|  | **Hm/Ms/Rt T1 snRNA** | CGACTGCATAATTTGTGGTAGTGG |
|  | **RNT43 snoRNA** | CTTATTGACGGGCGGACAGAAAC |
|  | **bta-miR-99b** | CACCCGTAGAACCGACCTTGCG |

**Supplementary Table S3. List of the 49 microRNAs detected in the epEVs.** The list of microRNAs detected in epEVs according to higher relative expression based on 2^-∆Cq^.

| **miRNA** | **Cq** | **∆Cq** | **2^-∆Cq^.** |
| --- | --- | --- | --- |
| bta-miR-935 | 17.398 | -18.950 | 506,414.514 |
| bta-miR-421 | 20.903 | -15.445 | 44,596.931 |
| bta-miR-654 | 20.909 | -15.439 | 44,426.449 |
| bta-miR-664b | 24.762 | -11.586 | 3,074.683 |
| bta-miR-1260b | 25.503 | -10.845 | 1,839.516 |
| bta-miR-335 | 26.803 | -9.545 | 747.164 |
| bta-miR-23b-3p | 27.702 | -8.646 | 400.676 |
| bta-miR-149-5p | 27.851 | -8.497 | 361.404 |
| bta-miR-425-3p | 28.749 | -7.599 | 193.920 |
| bta-miR-433 | 30.123 | -6.225 | 74.827 |
| bta-miR-431 | 31.247 | -5.101 | 34.321 |
| bta-miR-92b | 31.512 | -4.836 | 28.561 |
| bta-miR-411b | 31.856 | -4.492 | 22.507 |
| bta-miR-1281 | 31.885 | -4.463 | 22.056 |
| bta-miR-760-3p | 32.238 | -4.110 | 17.268 |
| bta-miR-1247-3p | 32.595 | -3.753 | 13.484 |
| bta-miR-568 | 32.677 | -3.671 | 12.737 |
| bta-miR-296-5p | 32.815 | -3.533 | 11.575 |
| bta-miR-450a | 33.176 | -3.172 | 9.014 |
| bta-miR-432 | 33.727 | -2.621 | 6.152 |
| bta-miR-219 | 33.976 | -2.372 | 5.175 |
| bta-miR-340 | 33.986 | -2.362 | 5.142 |
| bta-miR-542-5p | 34.342 | -2.006 | 4.016 |
| bta-miR-658 | 34.601 | -1.747 | 3.357 |
| bta-miR-342 | 34.685 | -1.663 | 3.167 |
| bta-miR-362-5p | 34.850 | -1.498 | 2.825 |
| bta-miR-665 | 34.886 | -1.462 | 2.754 |
| bta-miR-1246 | 34.942 | -1.406 | 2.650 |
| bta-miR-197 | 35.019 | -1.329 | 2.513 |
| bta-miR-27a-5p | 35.276 | -1.072 | 2.102 |
| bta-miR-758 | 35.661 | -0.687 | 1.610 |
| bta-miR-1224 | 35.692 | -0.656 | 1.575 |
| bta-miR-223 | 35.836 | -0.512 | 1.426 |
| bta-miR-182 | 35.945 | -0.403 | 1.322 |
| bta-miR-378b | 36.054 | -0.294 | 1.226 |
| bta-miR-376b | 36.094 | -0.254 | 1.193 |
| bta-miR-375 | 36.117 | -0.231 | 1.174 |
| bta-miR-424-3p | 36.153 | -0.195 | 1.145 |
| bta-miR-761 | 36.210 | -0.138 | 1.100 |
| bta-let-7b | 36.404 | 0.056 | 0.962 |
| bta-miR-574 | 36.485 | 0.137 | 0.909 |
| bta-let-7e | 36.488 | 0.140 | 0.908 |
| bta-miR-21-3p | 36.710 | 0.362 | 0.778 |
| bta-miR-1179 | 36.720 | 0.372 | 0.773 |
| bta-miR-940 | 36.766 | 0.418 | 0.749 |
| bta-miR-505 | 36.842 | 0.494 | 0.710 |
| bta-miR-339a | 36.894 | 0.546 | 0.685 |
| bta-miR-455-5p | 36.898 | 0.550 | 0.683 |
| bta-miR-129-5p | 36.954 | 0.606 | 0.657 |
| Hm/Ms/Rt T1 snRNA | 36.348 | - | - |

**Supplementary Table S4. Estimation of the physiological proportion between epididymosomes (epEVs) and sperm in bovine epididymal *cauda*.** Epididymal fluid collected from epididymis *cauda* by retrograde perfusion of six bulls were used to estimate the physiological proportion between epEVs and sperm. Following collection, epididymal fluid was processed to isolate the epEVs by ultracentrifugation. After isolation, epEVs were analyzed regarding concentration (Particles/mL) using the NanoSight^®^ NS300 and NanoSight^®^ Software 3.44 (NTA; Malvern Panalytical). From the collected epididymal fluid, sperm were also analyzed regarding sperm concentration. For that, a solution of 5μL of epididymal fluid diluted in 995μL of paraformaldehyde 4% in PBS was evaluated at a Neubauer chamber to estimate the concentration of sperm/mL. Following estimation of particles/mL and sperm/mL, the proportion of particles/sperm was calculated by dividing particles per sperm.

| **Sample** | **Particles/mL^1^** | **Sperm/mL^2^** | **Particles/sperm** |
| --- | --- | --- | --- |
| #1 | 20,10 x 10^9^ | 145.00 x 10^6^ | 138.62 |
| #2 | 31,80 x 10^9^ | 555.00 x 10^6^ | 57.30 |
| #3 | 73,40 x 10^9^ | 1,975.00 x 10^6^ | 37.16 |
| #4 | 92,90 x 10^9^ | 960.00 x 10^6^ | 96.77 |
| #5 | 60,50 x 10^9^ | 600.00 x 10^6^ | 100.83 |
| #6 | 29,10 x 10^9^ | 505.00 x 10^6^ | 57.62 |
| Total* | 51,30 x 10^9^ ± 11,76 x 10^9^ | 790.00 x 10^6^ ± 259.60 x 10^6^ | 81.38 ± 15.26 |

^1^NTA; ^2^Neubauer chamber; *Mean and SEM.

**Supplementary File S1. Dataset of predicted target transcripts and biological pathways associated with miR-935 and -421.** In the file, the target transcripts analyzed using the TargetScanHuman platform are included, as well as the KEGG pathways identified through DAVID analysis by listing the corresponding target transcripts.

**SUP FILE S1**
